# Diagnosing Fatty Liver Disease: A Comparative Evaluation of Metabolic Markers, Phenotypes, Genotypes and Established Biomarkers

**DOI:** 10.1371/journal.pone.0076813

**Published:** 2013-10-09

**Authors:** Sabine Siegert, Zhonghao Yu, Rui Wang-Sattler, Thomas Illig, Jerzy Adamski, Jochen Hampe, Susanna Nikolaus, Stefan Schreiber, Michael Krawczak, Michael Nothnagel, Ute Nöthlings

**Affiliations:** 1 Cologne Center for Genomics, University of Cologne, Cologne, Germany; 2 Institute of Experimental Medicine, Section of Epidemiology, Christian-Albrechts University Kiel, Kiel, Germany; 3 Institute of Epidemiology, Christian-Albrechts University Kiel, Kiel, Germany; 4 Research Unit of Molecular Epidemiology, Helmholtz-Zentrum München, Neuherberg, Germany; 5 Hannover Unified Biobank, Hannover Medical School, Hannover, Germany; 6 Genome Analysis Center, Institute of Experimental Genetics, Helmholtz-Zentrum München, Neuherberg, Germany; 7 Department of General Internal Medicine, University Hospital Schleswig-Holstein, Kiel, Germany; 8 Institute of Clinical Molecular Biology, Christian-Albrechts University Kiel, Kiel, Germany; 9 PopGen Biobank, University Hospital Schleswig-Holstein, Kiel, Germany; 10 Institute of Medical Informatics and Statistics, Christian-Albrechts University Kiel, Kiel, Germany; 11 Department of Nutrition and Food Sciences, Nutritional Epidemiology, University of Bonn, Bonn, Germany; University of Modena & Reggio Emilia, Italy

## Abstract

**Background:**

To date, liver biopsy is the only means of reliable diagnosis for fatty liver disease (FLD). Owing to the inevitable biopsy-associated health risks, however, the development of valid noninvasive diagnostic tools for FLD is well warranted.

**Aim:**

We evaluated a particular metabolic profile with regard to its ability to diagnose FLD and compared its performance to that of established phenotypes, conventional biomarkers and disease-associated genotypes.

**Methods:**

The study population comprised 115 patients with ultrasound-diagnosed FLD and 115 sex- and age-matched controls for whom the serum concentration was measured of 138 different metabolites, including acylcarnitines, amino acids, biogenic amines, hexose, phosphatidylcholines (PCs), lyso-PCs and sphingomyelins. Established phenotypes, biomarkers, disease-associated genotypes and metabolite data were included in diagnostic models for FLD using logistic regression and partial least-squares discriminant analysis. The discriminative power of the ensuing models was compared with respect to area under curve (AUC), integrated discrimination improvement (IDI) and by way of cross-validation (CV).

**Results:**

Use of metabolic markers for predicting FLD showed the best performance among all considered types of markers, yielding an AUC of 0.8993. Additional information on phenotypes, conventional biomarkers or genotypes did not significantly improve this performance. Phospholipids and branched-chain amino acids were most informative for predicting FLD.

**Conclusion:**

We show that the inclusion of metabolite data may substantially increase the power to diagnose FLD over that of models based solely upon phenotypes and conventional biomarkers.

## Introduction

Fatty liver disease (FLD) is a complex disease ranging from simple fat accumulation in the liver (steatosis) to fatty liver associated with inflammation (steatohepatitis). Non-alcoholic fatty liver disease (NAFLD), a sub-phenotype that is characterized by fat accumulation in the liver (>5% of the liver weight) in the absence of excessive alcohol intake (<20g per day), represents the most common form of chronic liver disease [1]. This notwithstanding, its etiology is not yet fully understood. NAFLD has been estimated to affect 20–30% of adults in Western societies [1,2], including Germany where the prevalence was reported to be as high as 30% [3]. Owing to different diagnostic criteria and different characteristics of the respective study populations (partially small samples and highly selected subjects), however, published estimates of NAFLD prevalence vary widely [1,4-8]. Alcoholic fatty liver disease (AFLD) as another sub-phenotype is associated with excessive alcohol consumption, and the prevalence of AFLD has recently been estimated to be three times lower than that of NAFLD. Nevertheless, despite their different etiologies, it is generally difficult to distinguish AFLD from NAFLD on the basis of morphological features alone [9]. To date, histological examination of liver tissue obtained at biopsy is the only reliable means to diagnose FLD, but its invasiveness and associated health risks as well as the high cost of the procedure [10] render liver biopsy unsuitable as a screening tool. On the other hand, currently available noninvasive imaging techniques such as ultrasound, magnetic resonance imaging or computer tomography have been criticized for a lack of sensitivity, high costs or high radiation exposure [1,11]. Routine laboratory analysis of biomarkers has also been proposed as a noninvasive alternative for FLD screening [12]. Alanine transaminase (ALT), known to be elevated in FLD patients, is the most commonly used biomarker in medical practice but has been criticized for poor sensitivity and specificity, too [1,7,11,12].

Extensive research on FLD diagnosis has been conducted in the past, and several prediction models based upon physical examination (particularly body mass index (BMI), waist and hip circumference) and biomarkers (triglycerides (TG), gamma glutamyl transpeptidase (GGT), ALT, aspartate transaminase (AST), glucose) have been proposed [13-18]. However, only Bedogni’s Fatty Liver Index (FLI), which combines BMI, waist circumference, GGT and TG, has become widely used in FLD diagnosis [19-24], although it has been criticized recently for yielding only fair agreement with ultrasonographic results [25] and yet has to be validated in external populations.

Since the liver is a metabolically active organ, metabolite concentrations are likely to change under FLD. We therefore set out to explore the potential benefit of using metabolite profiles to predict ultrasound-diagnosed FLD. We developed different models to predict the disease status based either upon phenotypes (e.g. anthropometric measures), conventional biomarkers (blood parameters), metabolic profiles or disease-associated genotypes, or upon combinations thereof and compared their diagnostic power against a model solely based on parameters included in Bedogni’s FLI [13].

## Materials and Methods

### Study design and study population

The present study was carried out in the PopGen control cohort, a population-based sample drawn from the city of Kiel, Northern Germany, between June 2005 and February 2006 [26]. Briefly, potential participants were selected at random from the local population registry and invited to visit the study center at the local university hospital. At baseline, participants donated a venous blood sample, completed a general questionnaire and underwent brief physical examination [27]. A total of 747 North German individuals (392 males, 355 females) were recruited. Participants were excluded from the present study if no serum sample was available (n=17), the participant could not be contacted for future studies (n=3) or information was missing on abdominal ultrasound (n=238), blood lipids (n=22), glycated hemoglobin (n=6), glucose (n=1), liver enzymes (n=3), waist or hip circumference (n=12), hypertension (n=7), physical strength (n=14) or medication use (n=1). A total of 116 individuals with, and 307 individuals without, ultrasound-diagnosed FLD were available for the study. For practicability reasons, 115 FLD patients were randomly chosen and complemented by 115 control individuals matched for sex and 5-year age groups (50 to 55, 56 to 60, …, 76 to 80).

### Ethics statement

All PopGen cohort members were of German descent and gave written informed consent prior to the study. All study procedures were approved by the ethics committee of the Medical Faculty of the Christian-Albrechts University, Kiel, Germany.

### Parameter assessment

Venous blood samples were collected at baseline and fasting serum samples were stored under quality-controlled conditions. All cohort members completed a self-administrated questionnaire on personal and family medical history, medication use, height and current weight, and on lifestyle factors such as smoking. At baseline, all PopGen participants underwent physical examination by a trained physician, including abdominal ultrasound, test of physical strength and measurement of waist and hip circumference and blood pressure. Information on alcohol consumption (C2-units per day) was obtained during an interview carried out by a physician. BMI and waist-to-hip ratio (WHR) were calculated from self-reported weight and height data, and from measured waist and hip circumference, respectively. Hypertension was defined either as systolic/diastolic blood pressure greater than 140/90 mm Hg or as previously diagnosed hypertension. Conventional biomarkers were determined either by an *ad hoc* analysis of plasma samples (total cholesterol, high-density lipoprotein (HDL), low-density lipoprotein (LDL), TG, glucose, hemoglobin, glycated hemoglobin, alkaline phosphatase, cholinesterase, GGT, ALT, AST) or in subsequent analyses of frozen serum aliquots (fetuin-A).

### Measurement of metabolites and quality control

The serum concentration of 186 metabolites was measured using the Absolute*IDQ* ^TM^ p180 Kit (BIOCRATES Life Sciences AG, Innsbruck, Austria), as previously described [28]. Detailed information on the assays and reagents used can be found in the Absolute*IDQ* ^TM^ p180 Kit manual (available at www.biocrates.com). Metabolite concentrations were measured on three different plates, each time with the same kit and the same set of three negative controls. Five additional positive controls (‘QC samples’) were included on each plate for further quality control. Only metabolites that had an average coefficient of variation <25% across the 15 QC samples, and a detection rate >90% in all 230 serum samples combined, were analyzed further. Detection thresholds (‘Limits of Detection’, LODs) for single metabolites were taken from the Analytical Specifications Absolute*IDQ* ^TM^ p180 Kit manual (AS-p180; available upon request at www.biocrates.com). After quality control, 138 metabolites remained for further analysis, including 14 acylcarnitines, 21 amino acids, 11 biogenic amines, 1 hexose, 68 phosphatidylcholines (PCs), 9 lyso-PCs and 14 sphingomyelins. The average coefficient of variation for those 138 metabolites was 9%. See Table S1 for detailed results of the performed quality control.

### Outcome assessment

The endpoint of interest in our primary analysis, FLD, was diagnosed by abdominal ultrasound with a GE Logiq3 sonographic instrument (General Electric Healthcare, Bedford, UK). In particular, imaging the liver and kidney by a longitudinal- or cross-sectional view was carried out by one of three trained physicians. The presence of FLD was defined as increased hyperechogenic ultrasound pattern of the liver to that of the kidney (bright liver). Since FLD is a complex disease, ranging from steatosis to steatohepatitis, and in view of the allegedly low sensitivity of ultrasound for FLD, we also employed sharper endpoint definitions in a sensitivity analysis. As previously reported, FLD is associated with increased ALT levels [7,11], obesity, type 2 diabetes and dyslipidemia (low levels of HDL and high levels of TG), rendering FLD the hepatic representation of the metabolic syndrome (MetS) [4,29]. More specifically, previous studies revealed the presence of at least one MetS feature in approximately 90% of NAFLD patients, and of three or more features in approximately one third of them [4,29]. A similarly increased prevalence of MetS and type 2 diabetes was also noted among patients with AFLD [30]. Therefore, more stringent endpoint definitions of FLD included an elevated ALT level (defined as exceeding the 75^th^ sex-specific percentile of the PopGen cohort, i.e. >30U/l in men and >25U/l in women) [31] and the presence of MetS features as defined by the International Diabetes Federation (IDF) (www.idf.org).

#### The genetic risk score (GRS)

Potential FLD susceptibility loci have been identified through candidate-gene studies and GWAS in the past [32-35]. Many of these studies focused upon FLD-related secondary end points such as increased ALT or AST levels, severity of steatosis, or fibrosis. Therefore, we considered only those genetic loci as suitable for further analysis that were identified in studies using NAFLD or nonalcoholic steatohepatitis (NASH) as the primary end point and that had their disease association confirmed in studies with the same endpoint. In total, we selected 14 single-nucleotide polymorphisms (SNPs) for inclusion in our study (Table S2). Genotype information was either available from public sources [36,37] or was obtained by imputation with Beagle (v. 3.3) [38]. Quality control was performed using R (v. 2.14.1) [39] and PLINK (v. 1.07) [40] (see Material S1 for details). After quality control, 187 samples (91 cases, 96 controls) and 10 SNPs (see Table S2) were available for further analysis. Instead of including each SNP individually in a given diagnostic model, we used a genetic risk score (GRS), calculated as the total number of risk alleles (‘allele dosage’) at the 10 SNPs. Note that, due to imputation of some genotypes, the allele dosage per SNP and therefore the GRS assumed fractional values for some samples and markers.

#### Statistical analysis

Group differences in non-metabolic characteristics were tested for statistical significance using a χ^2^ test for categorical variables and a Student’s *t* test for continuous variables. FLD status was predicted using logistic regression models based either upon phenotypes, conventional biomarkers, metabolic profiles or genotypes, or upon combinations thereof. Given partially strong correlations between some metabolites (Figure S1), instead of including each of those 138 markers separately metabolic profiles as obtained from partial least-squares discriminant analysis (PLS-DA) [41] were used in logistic regression models to avoid collinearity among predictors. In brief, PLS-DA focuses upon covariance maximization between predictors (metabolites) and response (FLD) when estimating the parameters of a linear regression model, not upon variance maximization of the predictors alone, and thus represents a regression extension of principal component analysis. Missing data for leucine (n=1), histamine (n=11), SDMA (n=9) and taurine (n=16) were imputed using the respective sample means of either cases or controls, as appropriate. For PLS-DA, all predictors were standardized to unit variance and zero mean. Leave-one-out cross-validation (CV) of a preliminary PLS-DA model, including all 138 metabolites and the whole study population (n=230), identified the first five PLS components as providing optimal discriminatory power (minimal root mean squared error of prediction: 46.6%). In subsequent comparative analyses, all logistic regression models based on metabolic profile information therefore included the first five PLS components derived from a PLS-DA model for all metabolic markers or a subset of them and the whole study population or a subset of it, as appropriate. The reference model in our analyses included those parameters that had already been combined in Bedogni’s FLI [13]. However, to allow for comparability to fuller diagnostic models, the prediction was based on regression coefficient estimates specific for the underlying study population instead of implementation of the crude FLI (i.e. equation with proposed regression coefficients). Since cases and controls were matched for sex and age, no adjustment for these covariates was necessary in the initial data analysis. However, matching was no longer ensured in subsequent subgroup analyses, stratified by genotype, alcohol consumption or more stringent endpoint definitions. For comparability with the initial analysis, we report only the results of the unadjusted subgroup analyses here. Subgroup analyses adjusted for sex and age gave similar results and did not change our conclusions (data not shown).

The area under curve (AUC) was calculated for each diagnostic model. P values were obtained by DeLong’s approach, comparing the AUC of potentially related predictive models [42]. Change in prediction performance was also quantified by the integrated discrimination improvement (IDI) measure [43]. This measure uses the difference, or ‘discrimination slope’, between the average probability among cases of being affected, as predicted by the model, and the average probability among controls of being affected, again following the model’s prediction. The difference (‘absolute IDI’) in as well as the ratio (‘relative IDI’) of the discrimination slopes of two models then serve as measures of discrimination improvement. We used 10-fold CV, with an equal number of cases and controls in each partition and a fixed classification threshold of 50%, to protect against an upward bias in AUC estimates that is likely to occur when using the same data set for both training and testing of prediction models [44]. Discriminative power of the PLS-DA was visualized as scatter plot of the first two PLS components for each metabolic profile. Moreover, the contribution of individual predictors to the PLS-DA model and, thus, to the derived PLS components was quantified by the ‘Variable Importance in the Projection (VIP)’ score [45]. With this approach, the average of the squared VIP scores equals unity, and predictors with a VIP score >1 are considered more important than others for discriminating between cases and controls.

Single metabolite concentrations were also analyzed by linear regression of log-transformed values, treating FLD status as the independent variable (0 unaffected, 1 affected) and adjusting for sex, age, BMI, MetS, TG, GGT, ALT and AST/ALT ratio. Metabolite concentrations were log-transformed to ensure a better fit to a Gaussian distribution because most metabolite distributions were right-skewed. In view of the strong correlation between metabolites, we used the Westfall and Young Step-Down MaxT procedure [46] to allow for multiple testing. P values below 0.05 were considered statistically significant.

All analyses were performed using the R statistical software (v. 2.14.1) [39]. For PLS-DA, we used R package *pls* (v. 2.3.0) [47]. ROC analysis was carried out using package *pROC* [48] and reclassification analysis was done using package *PredictABEL* [49].

## Results

### Study population

The present study comprised 115 FLD patients (44 female, 71 male) and 115 sex- and age-matched controls (Table 1). The mean age at the time of recruitment was 60 years in cases (range: 50-76 years) and 61 years in controls (50-77 years). As was to be expected, cases had significantly higher BMI (p=7.5×10^-11^) and WHR (p=1.5×10^-4^), higher levels of TG (p=0.005), GGT (p=0.002) and ALT (p=0.022), and substantially lower levels of HDL cholesterol (p=2.4×10^-5^) than controls. Likewise, significantly more cases than controls were also diagnosed with MetS (p=8.0×10^-4^). Information on alcohol use was available for 167 subjects only, and a significantly higher self-reported alcohol intake was observed among FLD patients (p=0.028).

**Table 1:** Study population characteristics.

			**Cases**	**Controls**	**p**
**Participant characteristics**					
	**N**		115	115	
	**Men** (n (%))		71 (62)	71 (62)	
	**Age**, years		60 (7)	61 (7)	0.800
		Range	50-76	50-77	
**Phenotypes**					
	**Body mass index**, kg/m^2^		28.64 (4.61)	24.92 (3.59)	7.52×10^-11^
		< 25 (n (%))	22 (19)	66 (57)	
		[25, 30) (n (%))	61 (53)	3 9 (34)	
		≥ 30 (n (%))	32 (28)	10 (9)	
	**Waist circumference**, cm		99.16 (12.81)	89.71 (12.36)	3.91×10^-8^
	**Hip circumference**, cm		107.42 (10.59)	101.89 (7.52)	8.52×10^-6^
	**Waist-to-hip ratio**		0.92 (0.08)	0.88 (0.09)	1.54×10^-4^
	**Ever smokers** (n (%))		73 (63)	62 (54)	0.181
	**Alcohol consumption** ^1^ (n (%))		46 (40)	36 (31)	0.311
		**Alcohol intake**, C2-units	0.77 (1.08)	0.46 (0.65)	0.028
	**Hypertension** (n (%))		80 (70)	64 (56)	0.041
	**Type 2 diabetes** (n (%))		6 (5)	3 (3)	0.355
	**Co-morbitities** ^2^ (n (%))				
		0	36 (31)	45 (39)	0.576
		1	45 (39)	46 (40)	
		2	24 (21)	18 (16)	
		3	8 (7)	5 (4)	
		4	2 (2)	1 (1)	
	**Metabolic syndrome** ^3^ (n (%))		51 (44)	26 (23)	7.98×10^-4^
	**Physical strength**, kg		37.99 (10.85)	37.10 (12.34)	0.561
	**Medication use** (n (%))		76 (66)	69 (60)	0.412
**Conventional biomarkers**					
	**Total cholesterol**, mg/dl		228.44 (61.35)	222 (40.34)	0.348
	**HDL cholesterol**, mg/dl		60.57 (16.42)	70.85 (19.55)	2.35×10^-5^
	**LDL cholesterol**, mg/dl		146.91 (39.81)	142.74 (34.84)	0.398
	**Triglyceride**, mg/dl		194.50 (295.27)	114.95 (50.45)	0.005
	**Fasting glucose**, mg/dl		91.21 (26.11)	86.20 (15.27)	0.077
	**Hemoglobin**, g/dl		14.88 (1.11)	14.51 (1.06)	0.009
	**Glycated hemoglobin**, %		5.74 (0.65)	5.58 (0.34)	0.016
	**Alkaline phosphatase**, U/l		73.37 (20.49)	71.19 (24.36)	0.463
	**Cholinesterase**, KU/l		9.45 (1.74)	8.99 (1.92)	0.058
	**Fetuin-A**, µg/ml		347.04 (89.31)	323.14 (93.86)	0.049
	**ALT**, U/l		31.36 (15.86)	25.26 (23.58)	0.022
	**AST**, U/l		25.46 (9.06)	25.68 (18.38)	0.910
	**GGT**, U/l		48.59 (47.88)	32.08 (29.12)	0.002
	**AST/ALT**		0.90 (0.26)	1.13 (0.28)	6.22×10^-10^
	**GGT/ALT**		1.62 (1.51)	1.38 (1.00)	0.163

Data are means (sd) unless indicated otherwise. P values were obtained from a χ^2^ or Wald test in a linear regression analysis of categorical and continuous predictors, respectively.

^1^ Based upon 167 individuals only because of missing data (29 cases, 34 controls).

^2^ Number of prevalent diseases, including cancer, chronic disease, any form of diabetes, gallstones, heart attack, inflammatory bowel disease and neuropathy.

^3^ The metabolic syndrome was defined according to the International Diabetes Federation (IDF) definition.

### Improved diagnostic accuracy when using the metabolic marker set, but little gain with genotype information

**Figure 1:**
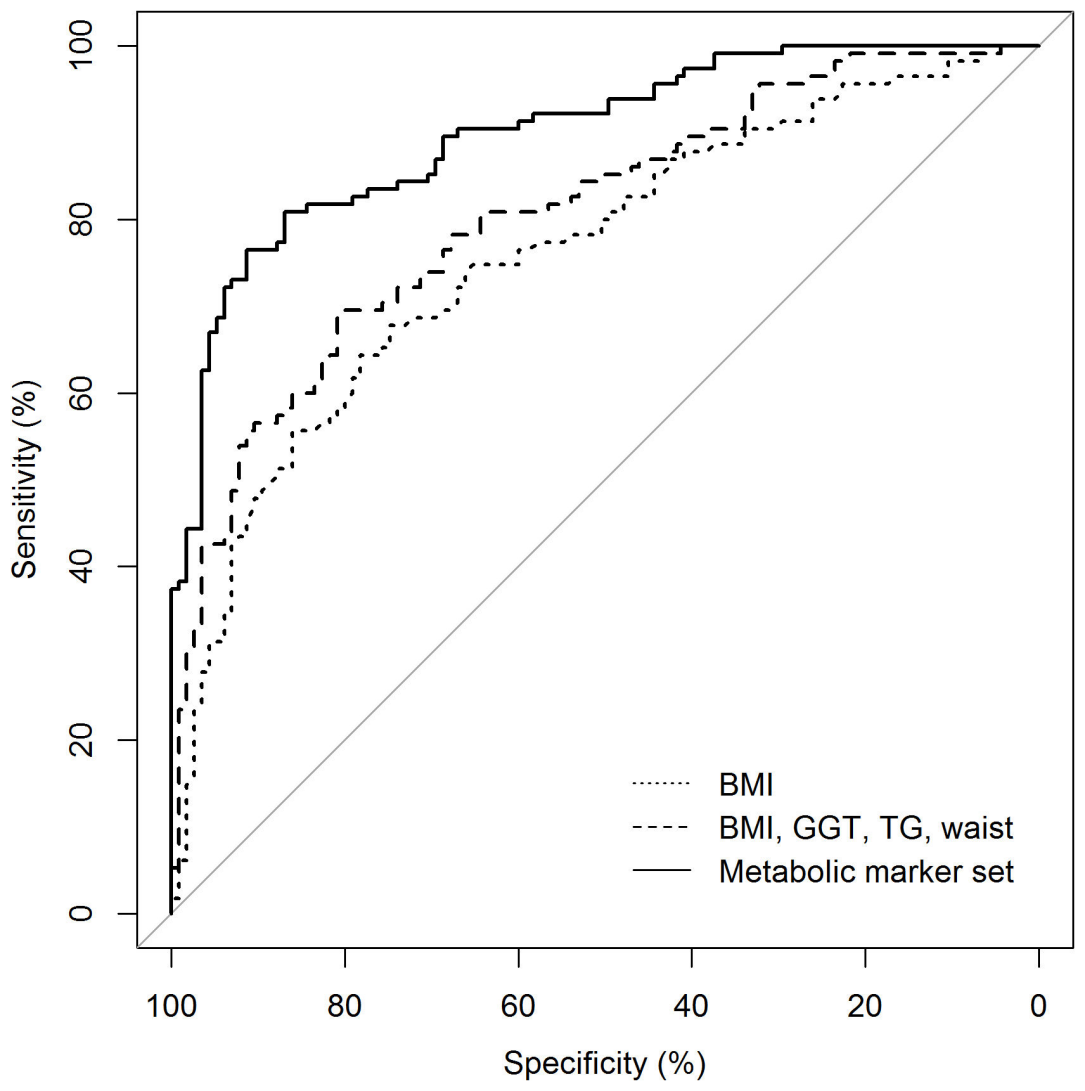
Comparison of the area under ROC curve (AUC) of three diagnostic models based upon 1: BMI; 2: BMI, GGT, TG, waist or 3: metabolic marker set. ROC statistics were based upon logistic regression analysis of 115 FLD patients and 115 controls. Compared to model 1 (AUC: 0.7609), AUC was significantly higher in both, model 2 (AUC: 0.8060, p=0.021) and model 3 (AUC: 0.8993, p=6.7×10^-6^). A significant increase was also noted from model 2 to 3 (p=4.2×10^-4^).

Prediction of FLD status in the baseline model based upon parameters included in Bedogni’s FLI (BMI, GGT, TG, waist circumference) achieved an AUC of 0.8060 (95% CI: [0.7503; 0.8616]; Table 2). Consideration of neither liver enzymes ALT and AST nor the whole set of phenotypes and biomarkers increased this value substantially (AUC: 0.8154, p=0.353 and AUC: 0.8375, p=0.052). However, a significantly higher AUC was noted when the first five PLS components from a PLS-DA model on 138 metabolites were included (AUC: 0.9135, p=1×10^-3^). Moreover, risk-based classification of individuals improved by an absolute IDI of 0.2471 (95% CI: [0.1917; 0.3025]), corresponding to a 90.0% increase of the difference between the mean risk of cases and controls. In addition, the classification error, as obtained from 10-fold CV, decreased to 20%. Again, inclusion of other single phenotypes and biomarkers did not substantially further improve the diagnostic accuracy. Notably however, BMI remained a significant predictor besides the PLS components in all prediction models (p<0.05; data not shown). Figure 1 depicts the diagnostic capability of BMI compared to that of BMI, GGT, TG and waist circumference and to that of the metabolic marker set, respectively. Remarkably, consideration of the first five PLS components alone decreased diagnostic accuracy of the model based additionally on parameters included in Bedogni’s FLI only marginally (AUC: 0.8993, p=0.125) reflecting high discriminative power of metabolic profiles independently from information provided by BMI, GGT, TG and waist circumference.

**Table 2:** Diagnostic accuracy for fatty liver disease.

				**ROC** ^1^			**IDI** ^2^		**Classification**
**Predictors**			**AUC**	**95% CI**	**p**	**Abs**	**95% CI**	**Rel**	**error** ^3^
**All study participants, n=230 (115 cases/ 115 controls)**									
		BMI, GGT, TG, waist (baseline)	0.8060	[0.7503; 0.8616]					29%
		BMI, GGT, TG, waist, ALT, AST	0.8154	[0.7611; 0.8697]	0.353	0.0223	[0.0028; 0.0417]	8.2%	30%
		Whole set of phenotypes^4^ and biomarkers^5^	0.8375	[0.7866; 0.8884]	0.052	0.0686	[0.0354; 0.1017]	25.2%	30%
		BMI, GGT, TG, waist, metabolites^6^	0.9135	[0.8784; 0.9486]	1.0×10^-3^	0.2471	[0.1917; 0.3025]	90.0%	20%
		BMI, GGT, TG, waist, ALT, AST, metabolites	0.9167	[0.8824; 0.9511]	7.5×10^-4^	0.2550	[0.1991; 0.3110]	93.8%	22%
		Whole set of phenotypes and biomarkers, metabolites	0.9233	[0.8901; 0.9564]	3.5×10^-7^	0.2824	[0.2249; 0.3399]	103.9%	22%
**Study participants with available genotype data, n=187 (91/96)**									
		BMI, GGT, TG, waist (baseline)	0.8023	[0.7406; 0.8640]					31%
		BMI, GGT, TG, waist, GRS^7^	0.8076	[0.7462; 0.8690]	0.550	0.0111	[-0.0035; 0.0257]	4.2%	32%
		BMI, GGT, TG, waist, ALT, AST	0.8053	[0.7441; 0.8665]	0.777	0.0143	[-0.0039; 0.0325]	5.3%	35%
		BMI, GGT, TG, waist, metabolites	0.9068	[0.8661; 0.9476]	2.9×10^-5^	0.2280	[0.1679; 0.2881]	85.3%	24%
		BMI, GGT, TG, waist, metabolites, GRS	0.9071	[0.8659; 0.9483]	2.9×10^-5^	0.2401	[0.1789; 0.3010]	89.8%	20%
		BMI, GGT, TG, waist, ALT, AST, metabolites	0.9097	[0.8699; 0.9495]	2.1×10^-5^	0.2314	[0.1709; 0.2919]	86.6%	24%
**Study participants with information on alcohol use, n=167 (86/81)**									
		BMI, GGT, TG, waist (baseline)	0.8246	[0.7627; 0.8864]					28%
		BMI, GGT, TG, waist, alcohol use	0.8262	[0.7647; 0.8876]	0.706	0.0026	[-0.0052; 0.0103]	-0.8%	26%
		BMI, GGT, TG, waist, ALT, AST	0.8285	[0.7675; 0.8894]	0.644	0.0101	[-0.0065; 0.0267]	3.2%	31%
		BMI, GGT, TG, waist, metabolites	0.9556	[0.9291; 0.9821]	2.8×10^-3^	0.3417	[0.2700; 0.4134]	108.4%	24%
		BMI, GGT, TG, waist, metabolites, alcohol use	0.9555	[0.9287; 0.9823]	3.6×10^-6^	0.3439	[0.2724; 0.4154]	109.1%	25%
		BMI, GGT, TG, waist, ALT, AST, metabolites	0.9591	[0.9341; 0.9841]	1.7×10^-6^	0.3534	[0.2812; 0.4256]	112.1%	26%

Diagnostic models are based upon logistic regression models.

^1^ Diagnostic models were evaluated by reference to AUC, the area under Receiver Operation Characteristics (ROCs) curves. P values were obtained by DeLong’s approach of comparing AUC between potentially related models.

^2^ Reclassification was assessed by the integrated discrimination improvement (IDI). The IDI is based upon the change, from one model to another, in terms of the so-called ‘discrimination slope’, defined as the difference in average FLD risk between cases and controls. Whereas the absolute IDI value of two models measures the classification improvement by the difference between their discrimination slopes, the relative IDI value equals the ratio of their discrimination slopes.

^3^ The performance of each diagnostic model was evaluated by 10-fold cross-validation with an equal number of cases and controls in each partition.

^4^ Phenotypes: sex, age, body mass index (BMI), waist circumference, hip circumference, smoking, hypertension, prevalent diseases (cancer, chronic disease, any form of diabetes, gallstones, heart attack, inflammatory bowel disease, neuropathy), physical strength, medication use.

^5^ Conventional biomarkers: HDL cholesterol, LDL cholesterol, triglycerides (TG), glucose, hemoglobin, glycated hemoglobin, alkaline phosphatase, cholinesterase, Fetuin-A, gamma-glutamyl transpeptidase (GGT), alanine transaminase (ALT), aspartate transaminase (AST).

^6^ Metabolites: the first five components derived from a partial least-squares analysis on 138 metabolites comprising 14 acylcarnitines, 21 amino acids, 11 biogenic amines, one hexose, 68 phosphatidylcholines (PCs), 9 lyso-PCs and 14 sphingomyelins

^7^ The genetic risk score (GRS) was calculated as the sum of risk allele dosages, thereby assuming an additive genetic model and an equal contribution to the risk of FLD for each of the 10 SNPs.

The use of genotype information (GRS) did not substantially improve the diagnostic accuracy of any model in a sensitivity analysis (Table 2). Moreover, inclusion of information on alcohol use, available for 167 participants (86 cases, 81 controls), did also not substantially improve AUC, IDI or the classification error of any of the models (Table 2). This result remained virtually unchanged when those seven FLD patients and one control were excluded who had reported an alcohol intake of more than 20g (>2 C2-units) per day (data not shown).

### Better diagnostic accuracy with sharper endpoint definitions

**Figure 2:**
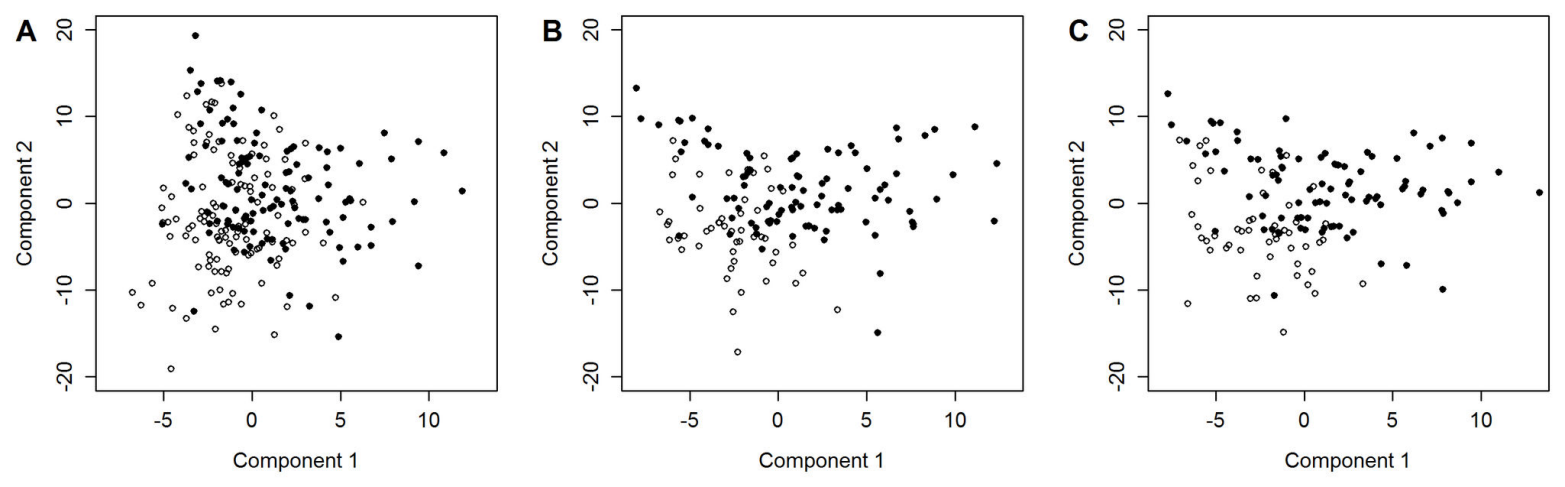
Scatter plots of the first two components of partial least-squares discriminant analyses (PLS-DA) of **fatty liver disease (FLD)**. FLD was defined (**A**) by ultrasound and more stringent (**B**) by ultrasound and elevated ALT level and (**C**) by ultrasound and the presence of the metabolic syndrome (MetS). PLS-DA components were obtained for 138 serum metabolite concentrations in (**A**) 230 individuals (115 cases, 115 controls), (**B**) 144 individuals (51 cases of fatty liver disease with elevated ALT level, 93 controls with normal ALT level) and (**C**) 140 individuals (51 cases of fatty liver disease and MetS, 89 controls without MetS), respectively. In the three models the first two components explained (**A**) 22.5% (16.1% and 6.4% respectively), (**B**) 42.4% (20.1% and 22.3%) and (**C**) 37.7% (18.7% and 19.0%) of the variation in the response (case-control status). **Open** **circles**: cases; **filled** **circles**: controls.

PLS components of the metabolite profiles allowed a moderate discrimination between cases and controls. In particular, the first two components explained 22.5% of the variation in FLD status and discriminated moderately between cases and controls (Figure 2A), whereas the first five components jointly explained 44.0% of variation in FLD status and 66.4% of the variation in the predictors (i.e. metabolite concentrations). Sharper endpoint definitions increased the ability to discriminate. More specific, since elevated ALT levels are known to accompany FLD, we choose to re-define FLD status in two ways, dependent upon (i) the ultrasound diagnosis of FLD and (ii) an elevated ALT level (>30U/l in men, >25U/l in women). First, FLD was deemed to be present when at least one of the two criteria was met and, second, when both criteria were met. A total of 51 study participants with and 22 without ultrasound-diagnosed FLD had elevated ALT levels (30 males, 21 females compared to 16 males, 6 females). Whilst the first endpoint definition did not improve prediction accuracy, AUC increased significantly from 0.8993 to 0.9686 (95% CI: [0.9449; 0.9923], p=0.003) when both diagnostic criteria were employed simultaneously (Table 3). Moreover, the classification error rate decreased to 11% and the first and second PLS components allowed notably better discrimination between cases and controls (Figure 2B).

**Table 3:** Diagnostic accuracy of 138 metabolites for different endpoint definitions of fatty liver disease, and for metabolic syndrome.

**Endpoint definition**		**ROC** ^1^			**Classification**
**Cases**	**Controls**	**AUC**	**95% CI**	**p**	**error** ^2^
US+^3^ (n=115)	no US+ (n=115)	0.8993	[0.8603; 0.9383]		20%
US+ or elevated ALT-level^4^ (n=137)	no US+ and no elevated ALT-level (n=93)	0.9159	[0.8793; 0.9524]	0.544	18%
US+ and elevated ALT-level (n=51)	no US+ and no elevated ALT-level (n=93)	0.9686	[0.9449; 0.9923]	0.003	11%
US+ and ≥ 1 trait of MetS^5^ (n=112)	no US+ (n=115)	0.9153	[0.8793; 0.9512]	0.554	19%
US+ and ≥ 2 traits of MetS (n=94)	no US+ (n=115)	0.9250	[0.8915; 0.9585]	0.328	18%
US+ and ≥ 3 traits of MetS (n=51)	no US+ (n=115)	0.9589	[0.9335; 0.9843]	0.012	14%
US+ and MetS (n=51)	no US+ and no MetS (n=89)	0.9883	[0.9766; 1.0000]	2.6×10^-5^	6%
MetS (n=77)	no MetS (n=153)	0.9431	[0.9108; 0.9755]	0.091	13%

^1^ Diagnostic models were evaluated by reference to AUC, the area under Receiver Operation Characteristics (ROCs) curves. Models were based upon a partial least-squares discrimination analysis of 138 metabolites. P values were obtained by DeLong’s approach of comparing AUC between a given model and the baseline model (baseline model: FLD was diagnosed by abdominal ultrasound).

^2^ The performance of each diagnostic model was evaluated by 10-fold cross-validation with an equal number of cases and controls in each partition.

^3^ US+ refers to FLD as diagnosed by abdominal ultrasound (i.e. defined as increased hyperechogenic ultrasound pattern of the liver).

^4^ Elevated alanine transaminase (ALT) level was defined as ALT>30U/l for men and ALT>25U/l for women.

^5^ The metabolic syndrome (MetS) was defined according to the International Diabetes Federation (IDF) definition.

Since FLD is regarded as the hepatic representation of the MetS, we also assessed endpoint definitions that included MetS features. The diagnostic accuracy was significantly improved when ultrasound-diagnosed FLD was combined with the presence of three or more MetS features (AUC: 0.9589, 95% CI: [0.9335; 0.9843], p=0.012). Diagnostic accuracy improved even further when participants with ultrasound-diagnosed FLD and MetS were to be distinguished from those lacking both criteria, as was evidenced by a significantly higher AUC (0.9883, 95% CI: [0.9766; 1.0000], p=2.6×10^-5^), lower 10-fold CV error rate (6%) and more pronounced separation in a PLS scatter plot (Figure 2C). No significant differences were noted between the two diagnostic models based upon either criterion alone (i.e. ultrasound-diagnosed FLD or MetS).

Adopting the sharper endpoint definitions, we also related the diagnostic accuracy of the metabolic profiles to that of phenotypes, conventional biomarkers and combinations thereof. With all combinations tested, the overall trend towards a significant improvement by the inclusion of the metabolic markers was confirmed (data not shown).

#### Single-metabolite analysis

As judged by the individual VIP scores from a PLS-DA, some metabolites turned out to discriminate better between cases and controls than others (Table S3). The most prominent ones (VIP>1.35) are summarized in Table 4. These markers included some lyso-PCs (particularly acyl C 17:0, C 18:1, C 18:2), PCs (particularly diacyl C 38:3 and C 40:5 and acyl-alkyl C 38:1, C 40:3 and C 38:2), the biogenic amine acetylornithine (Ac-Orn) and various amino acids (particularly leucine, isoleucine, citrulline and valine). See Table S3 for the loadings of single metabolites on the first five PLS components. Interestingly, the most predictive metabolites in PLS-DA were found to be independent of the respective endpoint definition. In particular, lyso-PCs acyl C 17:0, C 18:1 and C 18:2, PCs diacyl C 38:3 and C 40:5 as well as amino acids leucine, isoleucine and valine were consistently identified as the best discriminating metabolites in all models tested (VIP range: 1.10 to 1.76).

**Table 4:** Model parameter estimates for selected metabolites most prominent in single linear regression (p<0.05 adjusted model) and/or partial least-squares discriminant analysis (VIP≥1.35).

			**Linear regression on logarithmized metabolite concentrations^1^**					**VIP score^2^ from PLS-DA**
**Metabolite**			**Unadjusted model**			**Adjusted model**		****
			**β**	**p**		**β**	**p**	
Acylcarnitines								
	**C14:1**		0.05	0.417		0.17	0.006	0.47
	**C14:2**		0.06	0.365		0.20	0.010	0.72
	**C2**		0.10	0.053		0.16	0.008	1.11
**Amino Acids**								
	**Cit**		-0.01	0.821		0.09	0.127	1.38
	**Ile**		0.15	4.8×10^-4^		0.12	0.013	1.39
	**Leu^3^**		0.13	0.003		0.12	0.014	1.42
	**Tyr**		0.15	0.001		0.14	0.010	1.33
	**Val**		0.13	0.001		0.09	0.061	1.35
**Biogenic Amines**								
	**Ac-Orn**		-0.13	0.053		-0.05	0.506	1.42
**Phosphatidylcholines**								
	**PC aa C32:1**		0.23	0.001		0.23	0.004	1.31
	**PC aa C32:2**		0.10	0.119		0.21	0.007	0.82
	**PC aa C34:2**		0.06	0.130		0.13	0.007	0.99
	**PC aa C36:3**		0.11	0.031		0.17	0.008	0.98
	**PC aa C38:3**		0.18	0.001		0.15	0.020	1.66
	**PC aa C40:5**		0.13	0.020		0.10	0.139	1.36
	**PC ae C38:1**		-0.14	0.113		6.1×10^-4^	0.995	1.43
	**PC ae C38:2**		-0.08	0.200		0.04	0.584	1.37
	**PC ae C40:3**		-0.12	0.085		-0.03	0.706	1.42
**Lyso-Phosphatidylcholines**								
	**lysoPC a C17:0**		-0.18	0.004		-0.06	0.397	1.71
	**lysoPC a C18:1**		-0.11	0.057		-1.7×10^-3^	0.980	1.59
	**lysoPC a C18:2**		-0.12	0.061		0.04	0.581	1.44

^1^ P values and regression coefficients (β) derived from a linear regression analysis of the log-transformed metabolite concentrations in 230 study participants (115 FLD cases, 115 controls). All models were also adjusted for sex, age, BMI, TG level, hemoglobin, glycated hemoglobin and GGT.

^2^ VIP (Variable Importance in the Projection) scores were calculated on the basis of the first five components from partial least-squares discriminant analysis (PLS-DA) in 230 study participants. Missing values were imputed with corresponding sample means for cases or controls.

^3^ Linear regression analysis was based upon 229 individuals only because of missing data.

Linear regression analysis of log-transformed metabolite concentrations revealed a nominally significant association with FLD for 19 of the 138 metabolites (13.8%; Table S3). In view of the many phenotypic differences between cases and controls (Table 1), all models were also adjusted for sex, age, BMI, presence of MetS, TG, GGT, ALT and AST/ALT ratio. After adjustment, 24 of the 138 metabolites (17.4%) showed a nominally significant association with FLD, but none of them remained significant when multiple testing was allowed for (Table S3). The smallest nominal p values (p≤0.01) were obtained for PCs diacyl C 32:1 (p=0.004), C 32:2 (p=0.007), C 34:2 (p=0.007) and C 36:3 (p=0.008), for acylcarnitines tetradecenoyl-L-carnitine (C14:1, p=0.006), acetyl-L-carnitine (C2, p=0.008) and tetradecadienyl-L-carnitine (C14:2, p=0.01), and for amino acid tyrosine (p=0.01) (Table 4). Interestingly, almost half of the FLD associations of biogenic amines and phospholipids were reversed upon adjustment for covariates (Table S3).

Compared to metabolic profiles based upon the first five components from a PLS-DA model including all 138 metabolic markers, a very similar diagnostic accuracy was achieved when the five PLS components were derived from only those 59 metabolites that had VIP values above unity (AUC: 0.8848, 95% CI: [0.8431; 0.9264], p=0.135). Sparser models based upon either those 41 metabolites that had VIP>1.1 or those 24 metabolites that were significantly associated with FLD gave significantly lower discriminatory power (AUC: 0.8718, 95% CI: [0.8271; 0.9165], p=0.029; AUC: 75.96, 95% CI: [0.6987; 0.8205], p=2.6×10^-6^).

## Discussion

By comparing the diagnostic capability for FLD of different sets of predictors, including phenotypes, conventional biomarkers, metabolite profiles, genotypes, in a cross-sectional study, we could show that metabolic profiles yielded superior diagnostic accuracy among all sets. Moreover, inclusion of single phenotypes and biomarkers did not substantially improve the diagnostic accuracy over that of metabolites alone. This lack of improved accuracy may be due to the fact that the metabolite concentrations already captured most of the information provided by phenotypes and biomarkers. Inclusion of genotypic information did not contribute to the diagnostic accuracy of any prediction model.

### Comparison to existing diagnostic models

Past research on metabolism in FLD, especially on lipid metabolism, was aimed at the identification of biomarkers and a better understanding of the etiology of FLD [9,12,50-53]. In particular, two metabolome studies following an ‘untargeted approach’ revealed good discrimination between individuals with and without liver failure, based upon their metabolic profiles [12,52]. To our knowledge, however, our study is the first to compare the diagnostic capability of serum metabolic markers to that of established phenotypes, biomarkers and genotypes. Bedogni et al. [13] developed an algorithm for the prediction of FLD in the general population that is based on BMI, GGT, TG and waist circumference. Similar to our study, the authors had based their prediction tool upon ultrasound-diagnosed FLD. We could confirm diagnostic capability of parameters included in Bedogni’s FLI [13], with the exception of waist circumference, being not the strongest but the poorest predictor for FLD among those parameters in our study population. In addition, we could show that inclusion of metabolic makers can further increase accuracy for detecting FLD and, moreover, that this accuracy was not decreased substantially when the prediction model was based on metabolic profiles alone. Another fatty liver score derived by Kotronen et al. [14] suggested that the presence of the MetS and of type 2 diabetes, the levels of fasting serum insulin and AST and the AST/ALT ratio are independent predictors of FLD status. Moreover, Hamaguchi and colleagues recently reported an effective prediction of NAFLD from features of the MetS alone [54]. Even though MetS and AST/ALT ratio differed significantly between FLD patients and controls, however, the diagnostic accuracy was not improved notably by the inclusion of these potential predictors in our study. In line with our findings, Kotronen et al. also reported no significant improvement in diagnostic accuracy by the inclusion of genetic information [14]. Other algorithms to diagnose FLD incorporate different covariates from the ones considered here [15-18], thereby reflecting again the difficulty to select predictors for a generally useful diagnostic model for FLD.

### Improved diagnostic accuracy with sharper endpoint definitions

Metabolic markers showed the highest diagnostic accuracy in the present study. While the AUC value for the first five PLS-DA components equaled 0.8993, this likely represents an overestimate of the predictive power of metabolites for FLD status due to the use of the same data for both training and testing of the prediction model. Ten-fold CV yielded a more realistic and substantial classification error rate of 20% which corresponds to a misclassification of 47 of the 230 study participants. Such imprecise discrimination between cases and controls might be the consequence of a high variability of metabolite concentrations even in healthy subjects [55] and/or the low sensitivity and specificity of ultrasound as a diagnostic tool for FLD [1,11,56]. Indeed, sharper endpoint definitions of FLD resulted in a significant improvement of the diagnostic accuracy of the regression models and lower classification error rates throughout. The best discrimination was obtained for individuals with ultrasound-diagnosed FLD and MetS against those lacking both criteria. Metabolic profiles may therefore be useful for the classification of different subtypes of FLD as well but, unfortunately, no FLD subtype information was available in our study.

### Metabolites on FLD-related pathways?

VIP scores from a PLS-DA model and simple linear regression analysis identified several metabolites (amino acids, acylcarnitines, PCs) as possibly involved in one or more of the underlying metabolic pathways, rendering them promising candidates for diagnostic markers for FLD. In particular, the observed associations between FLD and PCs and acylcarnitines may, as was to be expected, point towards a disturbed lipid metabolism in FLD. Strong associations between FLD and alterations in phospholipid and amino acid metabolism have been already reported before [12,50-52] and are discussed elsewhere [57,58]. Higher levels of a given amino acid among cases (six amino acids with p<0.05) might reflect leakage from dying hepatocytes into the circulation [59]. Along the same line, Newgard et al. [60] observed significantly higher levels of amino acids and acylcarnitines in obese than in lean individuals (both without FLD). Since FLD is closely related to obesity, the possibility that our results reflect differences between lean and obese subjects rather than between FLD patients and controls therefore cannot be excluded. However, several obesity-related metabolites identified by Newgard et al. [60] were significantly associated with FLD in a linear regression analysis after adjustment for BMI, namely amino acids isoleucine, leucine and tyrosine and acylcarnitines C3 and C5. Branched-chain amino acids (BCAA) isoleucine and leucine, which are predominantly catabolized in extrahepatic tissue, and aromatic amino acid tyrosine could be of particular interest in future research because they are reportedly related to insulin resistance [60] and the development of type 2 diabetes [61]. In any case, additional experimental approaches are needed to characterize potential causal pathways.

### Limitations

Some limitations of our study should not go unmentioned. First, we used ultrasound for the definition of FLD, which cannot differentiate between histological subtypes of FLD and has been criticized for its low sensitivity [1,11,56]. However, since this is the first study investigating diagnostic capability of metabolite profiles for FLD, the study focus was on the ability of a metabolic marker set to detect FLD rather than to differentiate between single subtypes of FLD. In addition, despite criticism regarding possible misclassifications by ultrasonography, a recent meta-analysis [56] revealed that this screening method allows for reliable and accurate detection of moderate-severe FLD, compared to histology, making it currently the method of choice for FLD diagnosis in the general population [1]. Bedogni et al. [13] developed the FLI that may help physicians select subjects for liver ultrasonography and intensified lifestyle counseling. Our study suggests that the inclusion of metabolic markers could even further improve the accuracy to predict FLD status. Moreover, the diagnostic models based upon metabolic markers gave consistent results for different endpoint definitions and an improved diagnostic accuracy with increasing stringency. Given the evident ethical and practicability issues and associated risks of biopsies [11,13], a desirable investigation of the potential improvement of ultrasound diagnosis by incorporation of biomarker and especially metabolite profiles by comparison against the biopsy gold standard seems hardly feasible. Second, since our investigation of the impact of sharper endpoint definitions was partially based upon subgroups only, some of our results may have been due to data-overfitting because of small sample size. In any case, an improved diagnostic accuracy of metabolites was confirmed by lower error-rates obtained from 10-fold CV. Third, we are fully aware that a non-targeted metabolomics approach would have provided data on non-predetermined metabolites as well, some of them potentially related to FLD. However, the Absolute*IDQ* ^TM^ p180 Kit used in our study includes a panel of biologically relevant metabolites previously shown to be involved in main metabolic pathways. Moreover, targeted metabolomics allows a better interpretation of changes in single metabolite concentrations due to the availability of quantitative or semi-quantitative information. Fourth, information on several covariates (e.g. height, weight, alcohol use) was based on self-reports which always bear a risk of being imprecise. Moreover, information on alcohol use was available for only 167 subjects so that we could not distinguish *per se* between FLD with a nonalcoholic and an alcoholic etiology, both potentially associated with different metabolic profiles in FLD cases. In particular, excess alcohol use as a cause of FLD could not be ruled out in our seven cases that reportedly consumed more than 20g (>2 C2-units). However, since the ‘De Ritis Ratio’ of AST:ALT, proposed to distinguish between NAFLD and AFLD in the case of elevated levels of liver enzymes [62], among those 29 FLD patients lacking information on alcohol use in our study was <1 in the case of elevated ALT, AST and GGT levels, excessive alcohol intake turned out to be an unlikely cause of FLD in these patients. Moreover, our main results did not change qualitatively by the inclusion of alcohol use. We therefore surmise that the observed metabolic differences between FLD patients and controls were due to the presence of NAFLD-specific metabolite profiles. Finally, since serum metabolism reflects biochemical changes in many different tissues at a time, not only in a single tissue such as liver, our metabolic data may have been subjected to confounding by FLD-related co-morbidities. In addition, serum metabolite concentrations are known to be affected by factors like age, sex, genetic background, ethnicity, diurnal variation, diet, health status and physical activity level [63-65]. Anyhow, since cases and controls of our study were matched for sex and age, and moreover, came from the same confined geographical region of Northern Schleswig-Holstein, at least the possibility of confounding by sex and age as well as the risk of population bias, especially with regard to genotypic or metabolic markers, are likely to be negligible.

In conclusion, Bedogni’s FLI was proposed to help physicians to select individuals for liver ultrasonography and researchers to select patients for epidemiological studies. Our study revealed that a metabolic marker set formatted on a high-throughput platform also provides a high predictive accuracy for ultrasound-diagnosed FLD and exceeds the predictive power of that previously used set of predictors. Given the increasing interest in, and availability of, metabolic profiles in biomedical research, our results emphasize the need for an inclusion of metabolic markers into epidemiological and molecular studies of FLD, particularly also related to histological NAFLD. On the other hand, even although our study revealed a significantly better performance of metabolic markers compared to diagnostic models based upon phenotypes and conventional biomarkers alone, metabolite-based models are not sufficiently validated yet for use as diagnostic tools and ultrasonography remains an easier diagnosis tool at this time. Instead, we wish that our study stimulates further research into the diagnostic utility of metabolic profiles.

## Supporting Information

Table S1 **Results of performed quality control for the 186 metabolites.**
(PDF)

Table S2 **Previously reported SNPs associated with fatty liver disease.**
(PDF)

Table S3 **Mean metabolite concentrations for cases and controls and model parameter estimates from single linear regression and partial least-squares analysis.**
(PDF)

Material S1 **SNP genotyping, imputation and quality control.**
(PDF)

Figure S1 **Correlation structure among the 138 metabolites under study.** The graphical display was based on Pearson’s correlation coefficients. ACs: acylcarnitines (n=14), AAs: amino acids (n=21), BAs: biogenic amines (n=11), H: hexose (n=1), PCs: phosphatidylcholines (n=69), l-PCs: lyso-phosphatidylcholines (n=9), SMs: sphingomyelins (n=14)
(TIFF)
