# Supplementary material for: Diagnosing Fatty Liver Disease: A Comparative Evaluation of Metabolic Markers, Phenotypes, Genotypes and Established Biomarkers
Source: PLoS One. 2013 Oct 9;8(10):e76813. doi: 10.1371/journal.pone.0076813 (PMC3793954; doi:10.1371/journal.pone.0076813)
Supplement: Table S1 — Results of performed quality control for the 186 metabolites. (PDF) [file pone.0076813.s001.pdf]

Table S1 Results of performed quality control for the 186 metabolites

Metabolite	Coefficient of variation[1]	Proportion > LOD[2]	Number of missing values[3]
**Acylcarnitines**			
C0	7%	100%	0
C10	7%	93%	0
C10:1	9%	70%	0
C10:2	7%	48%	0
C12	6%	97%	0
C12-DC	5%	0%	0
C12:1	12%	19%	0
C14	10%	90%	0
C14:1	12%	100%	0
C14:1-OH	15%	50%	0
C14:2	15%	96%	0
C14:2-OH	16%	21%	0
C16	10%	100%	0
C16-OH	32%	6%	0
C16:1	29%	15%	0
C16:1-OH	27%	5%	0
C16:2	18%	55%	0
C16:2-OH	18%	0%	0
C18	11%	97%	0
C18:1	9%	100%	0
C18:1-OH	42%	3%	0
C18:2	11%	100%	0
C2	7%	100%	0
C3	7%	100%	0
C4-OH (C3-DC)	13%	6%	0
C3-OH	83%	40%	0
C3:1	24%	0%	0
C4	6%	100%	0
C4:1	12%	3%	0
C5	22%	100%	0
C5-DC (C6-OH)	56%	3%	0
C5-M-DC	19%	1%	0
C5-OH (C3-DC-M)	13%	0%	0
C5:1	11%	66%	0
C5:1-DC	16%	20%	0
C6 (C4:1-DC)	13%	42%	0
C6:1	20%	0%	0
C7-DC	14%	70%	0
C8	6%	81%	0
C9	29%	44%	0
**Amino Acids**			
Ala	13%	100%	0
Arg	9%	100%	0
Asn	15%	100%	0
Asp	20%	100%	0
Cit	15%	100%	0
Gln	9%	100%	0

Glu	14%	100%	0
Gly	10%	100%	0
His	11%	100%	0
Ile	8%	100%	0
Leu	8%	100%	1
Lys	14%	100%	0
Met	11%	100%	0
Orn	10%	100%	0
Phe	9%	100%	0
Pro	9%	100%	0
Ser	10%	100%	0
Thr	11%	100%	0
Trp	10%	100%	0
Tyr	9%	100%	0
Val	9%	100%	0
Biogenic Amines			
ADMA	17%	100%	0
Ac-Orn	6%	100%	0
Carnosine[4]	-	0%	229
Creatinine	12%	100%	0
DOPA	5%	34%	151
Dopamine[4]	-	0%	229
Histamine	5%	95%	11
Kynurenine	12%	100%	0
Met-SO	15%	100%	0
Nitro-Tyr[4]	-	0%	228
OH-Pro[4]	-	0%	220
PEA	4%	66%	79
Putrescine	26%	89%	2
SDMA	20%	95%	9
Sarcosine[4]	-	2%	225
Serotonin	30%	100%	0
Spermidine	23%	100%	0
Spermine[4]	-	10%	207
Taurine	13%	93%	16
alpha-AAA	9%	92%	0
total DMA	12%	100%	0
Hexoses			
H1	6%	100%	0
Phosphatidylcholines			
PC aa C24:0	33%	40%	0
PC aa C26:0	17%	0%	0
PC aa C28:1	11%	100%	0
PC aa C30:0	11%	100%	0
PC aa C30:2	199%	31%	0
PC aa C32:0	8%	100%	0
PC aa C32:1	9%	100%	0
PC aa C32:2	6%	100%	0
PC aa C32:3	9%	100%	0
PC aa C34:1	7%	100%	0
PC aa C34:2	5%	100%	0
PC aa C34:3	6%	100%	0
PC aa C34:4	6%	100%	0

**PC aa C36:0**	8%	100%	0
**PC aa C36:1**	7%	100%	0
**PC aa C36:2**	5%	100%	0
**PC aa C36:3**	5%	100%	0
**PC aa C36:4**	5%	100%	0
**PC aa C36:5**	6%	100%	0
**PC aa C36:6**	6%	100%	0
**PC aa C38:0**	7%	100%	0
**PC aa C38:1**	13%	100%	0
**PC aa C38:3**	6%	100%	0
**PC aa C38:4**	4%	100%	0
**PC aa C38:5**	5%	100%	0
**PC aa C38:6**	6%	100%	0
**PC aa C40:1**	11%	52%	0
**PC aa C40:2**	14%	100%	0
**PC aa C40:3**	10%	100%	0
**PC aa C40:4**	5%	100%	0
**PC aa C40:5**	5%	100%	0
**PC aa C40:6**	5%	100%	0
**PC aa C42:0**	9%	100%	0
**PC aa C42:1**	10%	100%	0
**PC aa C42:2**	10%	100%	0
**PC aa C42:4**	10%	100%	0
**PC aa C42:5**	10%	100%	0
**PC aa C42:6**	6%	98%	0
**PC ae C30:0**	16%	100%	0
**PC ae C30:1**	110%	80%	0
**PC ae C30:2**	19%	0%	0
**PC ae C32:1**	9%	100%	0
**PC ae C32:2**	11%	100%	0
**PC ae C34:0**	8%	100%	0
**PC ae C34:1**	8%	100%	0
**PC ae C34:2**	8%	100%	0
**PC ae C34:3**	7%	100%	0
**PC ae C36:0**	16%	100%	0
**PC ae C36:1**	6%	100%	0
**PC ae C36:2**	6%	100%	0
**PC ae C36:3**	6%	100%	0
**PC ae C36:4**	6%	100%	0
**PC ae C36:5**	6%	100%	0
**PC ae C38:0**	8%	100%	0
**PC ae C38:1**	9%	100%	0
**PC ae C38:2**	8%	100%	0
**PC ae C38:3**	5%	100%	0
**PC ae C38:4**	6%	100%	0
**PC ae C38:5**	6%	100%	0
**PC ae C38:6**	7%	100%	0
**PC ae C40:1**	9%	100%	0
**PC ae C40:2**	7%	100%	0
**PC ae C40:3**	7%	100%	0
**PC ae C40:4**	7%	100%	0
**PC ae C40:5**	5%	100%	0
**PC ae C40:6**	6%	100%	0

PC ae C42:0	8%	78%	0
PC ae C42:1	12%	100%	0
PC ae C42:2	9%	100%	0
PC ae C42:3	8%	100%	0
PC ae C42:4	7%	97%	0
PC ae C42:5	5%	87%	0
PC ae C44:3	13%	100%	0
PC ae C44:4	11%	100%	0
PC ae C44:5	7%	100%	0
PC ae C44:6	6%	100%	0
**Lyso-Phosphatidylcholines**			
lysoPC a C14:0	6%	70%	0
lysoPC a C16:0	7%	100%	0
lysoPC a C16:1	8%	100%	0
lysoPC a C17:0	8%	100%	0
lysoPC a C18:0	6%	100%	0
lysoPC a C18:1	7%	100%	0
lysoPC a C18:2	7%	100%	0
lysoPC a C20:3	8%	100%	0
lysoPC a C20:4	7%	100%	0
lysoPC a C24:0	24%	0%	0
lysoPC a C26:0	36%	4%	0
lysoPC a C26:1	7%	0%	0
lysoPC a C28:0	23%	47%	0
lysoPC a C28:1	25%	99%	0
**Sphingomyelins**			
SM (OH) C14:1	10%	100%	0
SM (OH) C16:1	9%	100%	0
SM (OH) C22:1	9%	100%	0
SM (OH) C22:2	9%	100%	0
SM (OH) C24:1	10%	100%	0
SM C16:0	9%	100%	0
SM C16:1	8%	100%	0
SM C18:0	8%	100%	0
SM C18:1	8%	100%	0
SM C20:2	10%	100%	0
SM C22:3[4]	-	0%	0
SM C24:0	9%	100%	0
SM C24:1	10%	100%	0
SM C26:0	19%	100%	0
SM C26:1	15%	100%	0

A red value indicates that the metabolite didn't met the quality control criteria (CV or LOD). Metabolites with blue background were excluded from the present study.

[1] average coefficient of variation (CV) across the 15 QC samples

[2] proportion of metabolites above the detection threshold (‘Limits of Detection’, LOD)

[3] total number of samples where serum concentration could not be determined

[4] serum concentration could not be determined for any of the 15 QC samples
