# Supplementary material for: Diagnosing Fatty Liver Disease: A Comparative Evaluation of Metabolic Markers, Phenotypes, Genotypes and Established Biomarkers
Source: PLoS One. 2013 Oct 9;8(10):e76813. doi: 10.1371/journal.pone.0076813 (PMC3793954; doi:10.1371/journal.pone.0076813)
Supplement: Table S2 — Previously reported SNPs associated with fatty liver disease. (PDF) [file pone.0076813.s002.pdf]

Table S2 Previously reported SNPs associated with fatty liver disease

Chromosome	SNP ID	Mapped Gene	Risk Allele	Reference
1	rs1801131	MTHFR	C	[1-2]
1	rs12137855*	LYPLAL1	C	[2]
2	rs780094	GCKR	T	[2]
2	rs16944	IL1B	T	[1-3]
4	rs6843722	CLOCK	C	[2]
6	rs361525	TNF-α	A	[1-3]
7	rs1800795	IL6	C	[1-2]
8	rs4240624	PPP1R3B	A	[2]
8	rs2645424	FDFT1	A	[2]
11	rs2854116*	APOC3	T	[2]
12	rs767870	ADIPOR2	T	[1-2]
17	rs7946	PEMT	A	[1-3]
19	rs2228603*	NCAN	T	[2]
22	rs738409*	PNPLA3	G	[1-2]

* SNPs which did not pass the quality controls prior to our analysis.

1 Anstee QM, Daly AK, Day CP (2011) Genetics of alcoholic and nonalcoholic fatty liver disease. Semin Liver Dis 31: 128-146.

2 Hernaez R (2011) Genetic factors associated with the presence and progression of nonalcoholic fatty liver disease: A narrative

3 Osterreicher CH, Brenner DA (2007) The genetics of nonalcoholic fatty liver disease. Ann Hepatol 6: 83-88.
