# Supplementary material for: Diagnosing Fatty Liver Disease: A Comparative Evaluation of Metabolic Markers, Phenotypes, Genotypes and Established Biomarkers
Source: PLoS One. 2013 Oct 9;8(10):e76813. doi: 10.1371/journal.pone.0076813 (PMC3793954; doi:10.1371/journal.pone.0076813)
Supplement: Table S3 — Mean metabolite concentrations for cases and controls and model parameter estimates from single linear regression and partial least-squares analysis. (PDF) [file pone.0076813.s003.pdf]

Table S3 Mean metabolite concentrations for cases and controls and model parameter estimates from single linear regression and partial least-squares analysis

Metabolite	Absolute metabolite concentrations (mean ± sd)		Linear regression on logarithmized metabolite concentrations[1]					Partial least-square discriminant analysis[3]					
	Cases (n=115) (µmol/l)	Controls (n=115) (µmol/l)	Unadjusted model		Adjusted model			VIP score	Loadings				
			β	p	β	p	p_{adj}[2]		Comp 1	Comp 2	Comp 3	Comp 4	Comp 5
**Acylcarnitines**													
**C0**	36,80 ± 10,28	33,27 ± 9,36	0,10	0,016	0,09	0,058	0,359	1,17	-0,0029	-0,0028	-0,0026	-0,0027	0,0042
**C10**	0,35 ± 0,16	0,35 ± 0,21	0,04	0,548	0,14	0,055	0,354	0,63	-1,41E-05	-0,0004	0,0026	0,0007	0,0053
**C12**	0,15 ± 0,07	0,15 ± 0,07	0,04	0,468	0,17	0,011	0,129	0,53	-0,0004	-0,0007	0,0016	-0,0005	0,0014
**C14**	0,05 ± 0,02	0,05 ± 0,02	0,02	0,639	0,13	0,019	0,181	0,57	-0,0001	-0,0006	0,0021	0,0027	0,0025
**C14:1**	0,15 ± 0,05	0,14 ± 0,06	0,05	0,417	0,17	0,006	0,079	0,47	-0,0004	-0,0010	4,58E-05	-0,0014	-0,0022
**C14:2**	0,03 ± 0,02	0,03 ± 0,02	0,06	0,365	0,20	0,010	0,115	0,72	-0,0009	-0,0013	-0,0016	-0,0062	-0,0048
**C16**	0,14 ± 0,05	0,13 ± 0,05	0,06	0,232	0,12	0,053	0,345	0,70	-0,0011	-0,0015	0,0007	0,0008	0,0011
**C18**	0,05 ± 0,02	0,05 ± 0,02	-0,01	0,915	0,04	0,474	0,833	0,90	0,0005	-0,0002	0,0041	0,0045	0,0097
**C18:1**	0,16 ± 0,06	0,15 ± 0,06	0,06	0,259	0,11	0,056	0,355	0,65	-0,0011	-0,0015	0,0004	-0,0005	0,0007
**C18:2**	0,06 ± 0,03	0,06 ± 0,02	0,07	0,216	0,16	0,017	0,168	0,86	-0,0014	-0,0018	-0,0013	-0,0059	-0,0051
**C2**	8,21 ± 3,08	7,47 ± 3,08	0,10	0,053	0,16	0,008	0,102	1,11	-0,0020	-0,0023	-0,0054	-0,0097	-0,0088
**C3**	0,38 ± 0,16	0,33 ± 0,13	0,16	0,003	0,12	0,049	0,334	1,34	-0,0030	-0,0026	-0,0003	0,0024	0,0097
**C4**	0,19 ± 0,09	0,17 ± 0,11	0,13	0,031	0,09	0,181	0,633	0,71	-0,0016	-0,0016	-0,0006	-0,0023	0,0013
**C5**	0,15 ± 0,06	0,13 ± 0,05	0,14	0,010	0,13	0,029	0,236	1,14	-0,0030	-0,0028	-0,0036	-0,0050	0,0004
**Amino Acids**													
**Ala**	424,85 ± 116,41	407,04 ± 138,83	0,06	0,145	0,05	0,328	0,772	0,88	-0,0011	-0,0012	0,0027	0,0003	0,0038
**Arg**	111,59 ± 35,73	114,04 ± 39,21	-0,01	0,873	0,04	0,514	0,794	0,66	0,0005	-0,0003	0,0027	0,0023	0,0056
**Asn**	46,47 ± 13,08	46,04 ± 13,78	0,01	0,744	0,06	0,161	0,606	1,11	-0,0003	-0,0005	0,0057	0,0020	0,0055
**Asp**	41,30 ± 14,42	39,17 ± 12,98	0,05	0,353	0,06	0,295	0,753	0,90	-0,0013	-0,0014	0,0004	-0,0049	0,0010
**Cit**	32,11 ± 10,63	32,91 ± 13,13	-0,01	0,821	0,09	0,127	0,542	1,38	0,0005	2,69E-05	0,0034	-0,0071	-0,0120
**Gln**	457,70 ± 128,61	463,80 ± 131,63	-0,01	0,845	0,07	0,117	0,524	1,04	0,0004	-0,0002	0,0055	0,0005	-0,0003
**Glu**	135,82 ± 55,50	120,72 ± 56,43	0,13	0,034	0,06	0,325	0,776	0,92	-0,0022	-0,0020	-0,0003	-0,0023	0,0012
**Gly**	244,89 ± 68,31	261,95 ± 79,29	-0,06	0,148	0,01	0,823	0,840	1,12	0,0019	0,0010	0,0069	0,0030	0,0031
**His**	82,08 ± 19,64	80,93 ± 21,22	0,02	0,549	0,06	0,153	0,588	0,89	-0,0005	-0,0009	0,0035	0,0032	0,0064
**Ile**	75,54 ± 22,67	65,26 ± 22,12	0,15	4,83E-04	0,12	0,013	0,139	1,39	-0,0036	-0,0033	-0,0037	-0,0047	0,0018
**Leu**[4]	169,89 ± 53,11	148,08 ± 44,98	0,13	0,003	0,12	0,014	0,150	1,42	-0,0035	-0,0034	-0,0051	-0,0081	-0,0014
**Lys**	154,69 ± 41,68	147,19 ± 42,17	0,05	0,177	0,05	0,257	0,724	1,18	-0,0015	-0,0015	0,0038	0,0051	0,0086
**Met**	19,78 ± 5,39	18,53 ± 5,28	0,07	0,081	0,10	0,023	0,205	1,04	-0,0019	-0,0018	0,0020	-0,0005	0,0014
**Orn**	71,62 ± 22,55	65,99 ± 21,08	0,08	0,072	0,13	0,016	0,165	1,14	-0,0021	-0,0021	-0,0006	-0,0066	-0,0020
**Phe**	81,06 ± 23,10	76,09 ± 22,72	0,07	0,116	0,08	0,137	0,558	0,85	-0,0018	-0,0021	-0,0014	-0,0034	0,0015
**Pro**	181,19 ± 55,50	167,48 ± 64,04	0,10	0,029	0,12	0,020	0,182	1,01	-0,0019	-0,0020	-0,0033	-0,0087	-0,0056
**Ser**	126,46 ± 38,28	132,27 ± 43,37	-0,03	0,495	0,03	0,549	0,803	1,09	0,0012	0,0003	0,0055	0,0033	0,0101
**Thr**	115,08 ± 31,79	119,28 ± 45,69	-0,01	0,847	0,08	0,121	0,531	1,27	0,0009	0,0005	0,0075	0,0012	-0,0009

**Trp**	54,73 ± 18,46	50,88 ± 17,41	0,08	0,122	0,10	0,108	0,501	0,95	-0,0017	-0,0020	0,0007	-0,0021	-0,0003
**Tyr**	67,38 ± 20,95	58,24 ± 20,68	0,15	0,001	0,14	0,010	0,115	1,33	-0,0035	-0,0033	-0,0042	-0,0062	-0,0037
**Val**	210,00 ± 57,43	184,99 ± 55,97	0,13	0,001	0,09	0,061	0,368	1,35	-0,0035	-0,0033	-0,0043	-0,0044	0,0025
**Biogenic Amines**													
**ADMA**	0,44 ± 0,13	0,45 ± 0,16	-1,80E-03	0,971	0,03	0,566	0,805	1,32	0,0005	0,0001	0,0082	0,0076	0,0076
**Ac-Orn**	0,77 ± 0,41	0,92 ± 0,75	-0,13	0,053	-0,05	0,506	0,792	1,42	0,0020	0,0016	0,0094	0,0103	0,0008
**Creatinine**	75,78 ± 20,05	75,80 ± 26,51	0,01	0,721	0,05	0,222	0,683	1,28	4,82E-06	-0,0002	0,0047	-0,0047	-0,0064
**Histamine**[4]	0,51 ± 0,03	0,51 ± 0,03	2,50E-03	0,743	0,01	0,475	0,816	0,91	-0,0004	-0,0007	-0,0045	-0,0091	-0,0016
**Kynurenine**	2,86 ± 0,88	2,77 ± 1,04	0,05	0,271	0,05	0,284	0,747	1,27	-0,0007	-0,0007	0,0065	0,0083	0,0078
**Met-SO**	1,64 ± 0,90	1,67 ± 1,04	-3,90E-03	0,956	0,15	0,074	0,413	1,11	0,0002	-0,0002	0,0062	0,0076	0,0074
**SDMA**[4]	0,66 ± 0,17	0,69 ± 0,20	-0,03	0,466	0,03	0,456	0,828	1,11	0,0011	0,0008	0,0076	0,0054	0,0011
**Spermidine**	0,33 ± 0,10	0,33 ± 0,09	-4,00E-03	0,917	0,01	0,855	0,845	0,77	-4,51E-05	-0,0004	-0,0006	-0,0048	0,0056
**Taurine**[4]	156,11 ± 50,84	155,11 ± 57,86	0,02	0,673	0,11	0,091	0,460	0,78	-0,0002	-0,0007	0,0012	-0,0048	-0,0019
**alpha-AAA**	0,70 ± 0,21	0,71 ± 1,05	0,11	0,029	0,04	0,506	0,795	0,75	0,0001	0,0003	0,0039	0,0073	0,0064
**total DMA**	1,00 ± 0,24	1,03 ± 0,29	-0,01	0,809	0,07	0,115	0,520	1,16	0,0007	1,29E-06	0,0050	-0,0023	-0,0062
**Hexoses**													
**H1**	4428,20 ± 1483,72	4163,25 ± 1147,22	0,06	0,141	0,06	0,173	0,623	0,73	-0,0016	-0,0018	-0,0008	-0,0007	0,0028
**Phosphatidylcholines**													
**PC aa C28:1**	3,24 ± 1,12	3,32 ± 1,30	-0,01	0,917	0,07	0,279	0,741	0,59	0,0005	-0,0007	-0,0017	-0,0028	-0,0050
**PC aa C30:0**	5,11 ± 1,66	5,20 ± 2,39	0,03	0,564	0,12	0,053	0,345	0,71	0,0004	-0,0005	0,0012	0,0054	0,0010
**PC aa C32:0**	14,03 ± 4,24	13,85 ± 4,74	0,03	0,505	0,09	0,088	0,454	0,61	-0,0003	-0,0013	-0,0014	0,0016	0,0006
**PC aa C32:1**	18,37 ± 9,09	15,26 ± 8,27	0,23	0,001	0,23	0,004	0,061	1,31	-0,0029	-0,0028	-0,0048	0,0020	-0,0025
**PC aa C32:2**	4,11 ± 1,50	4,03 ± 2,15	0,10	0,119	0,21	0,007	0,095	0,82	-0,0004	-0,0012	-0,0028	-0,0040	-0,0112
**PC aa C32:3**	0,47 ± 0,16	0,51 ± 0,20	-0,05	0,319	0,04	0,554	0,801	0,74	0,0017	0,0002	0,0004	0,0008	-0,0027
**PC aa C34:1**	216,81 ± 64,36	203,75 ± 66,82	0,08	0,073	0,12	0,030	0,239	0,91	-0,0016	-0,0022	-0,0032	0,0015	0,0012
**PC aa C34:2**	353,87 ± 85,53	339,97 ± 103,15	0,06	0,130	0,13	0,007	0,094	0,99	-0,0012	-0,0021	-0,0051	-0,0081	-0,0094
**PC aa C34:3**	16,84 ± 6,10	16,27 ± 7,40	0,08	0,175	0,17	0,013	0,143	0,71	-0,0007	-0,0016	-0,0040	-0,0025	-0,0059
**PC aa C34:4**	2,02 ± 0,79	1,92 ± 0,99	0,11	0,097	0,11	0,133	0,551	0,99	-0,0009	-0,0014	-0,0001	0,0051	-0,0052
**PC aa C36:0**	3,00 ± 1,16	3,19 ± 1,28	-0,05	0,389	2,40E-03	0,971	0,784	0,71	0,0013	-0,0002	-0,0019	0,0002	-0,0006
**PC aa C36:1**	52,70 ± 18,45	49,46 ± 19,41	0,09	0,094	0,11	0,087	0,448	0,84	-0,0014	-0,0022	-0,0048	-0,0019	-0,0017
**PC aa C36:2**	241,08 ± 68,03	231,59 ± 78,79	0,07	0,170	0,12	0,036	0,267	1,04	-0,0010	-0,0020	-0,0055	-0,0091	-0,0097
**PC aa C36:3**	136,45 ± 40,20	126,94 ± 47,66	0,11	0,031	0,17	0,008	0,102	0,98	-0,0017	-0,0025	-0,0052	-0,0058	-0,0081
**PC aa C36:4**	186,03 ± 58,32	174,72 ± 61,32	0,08	0,101	0,08	0,162	0,605	1,06	-0,0015	-0,0019	0,0004	0,0061	0,0017
**PC aa C36:5**	37,30 ± 24,17	33,22 ± 18,40	0,09	0,200	0,06	0,495	0,818	1,13	-0,0015	-0,0019	-0,0031	0,0050	-0,0013
**PC aa C36:6**	1,30 ± 0,62	1,31 ± 0,66	0,02	0,737	0,04	0,615	0,822	0,93	4,25E-05	-0,0010	-0,0030	0,0032	-0,0042
**PC aa C38:0**	3,14 ± 1,29	3,32 ± 1,40	-0,05	0,445	-2,61E-03	0,971	0,810	0,59	0,0011	-0,0003	-0,0010	-0,0017	-0,0027
**PC aa C38:1**	1,29 ± 0,51	1,40 ± 0,56	-0,07	0,262	-0,02	0,781	0,828	0,87	0,0016	-3,31E-05	-0,0029	-0,0014	0,0015
**PC aa C38:3**	53,60 ± 19,13	45,50 ± 16,38	0,18	0,001	0,15	0,020	0,185	1,66	-0,0036	-0,0039	-0,0100	-0,0088	-0,0097
**PC aa C38:4**	103,81 ± 36,91	94,26 ± 36,91	0,11	0,043	0,06	0,372	0,801	1,16	-0,0021	-0,0023	-0,0008	0,0055	0,0016
**PC aa C38:5**	60,87 ± 24,02	57,42 ± 21,68	0,06	0,273	0,04	0,489	0,823	1,32	-0,0012	-0,0017	0,0003	0,0098	0,0043

**PC aa C38:6**	92,69 ± 39,96	88,59 ± 37,02	0,04	0,490	0,05	0,497	0,817	0,73	-0,0009	-0,0015	-0,0018	0,0028	-0,0003
**PC aa C40:2**	0,40 ± 0,21	0,47 ± 0,31	-0,09	0,194	-0,03	0,714	0,859	1,30	0,0021	0,0005	-0,0053	-0,0029	0,0024
**PC aa C40:3**	0,67 ± 0,26	0,73 ± 0,35	-0,05	0,432	0,01	0,855	0,834	1,17	0,0016	-0,0001	-0,0052	-0,0015	0,0037
**PC aa C40:4**	3,50 ± 1,35	3,19 ± 1,29	0,12	0,042	0,10	0,128	0,539	1,03	-0,0019	-0,0023	-0,0037	0,0025	0,0013
**PC aa C40:5**	11,80 ± 4,67	10,41 ± 4,06	0,13	0,020	0,10	0,139	0,562	1,36	-0,0026	-0,0028	-0,0037	0,0047	0,0009
**PC aa C40:6**	33,36 ± 14,85	29,97 ± 12,67	0,10	0,095	0,05	0,491	0,821	1,05	-0,0020	-0,0024	-0,0046	0,0013	-0,0001
**PC aa C42:0**	0,53 ± 0,20	0,58 ± 0,24	-0,08	0,148	3,45E-03	0,959	0,813	0,94	0,0020	0,0004	-0,0006	-0,0041	-0,0065
**PC aa C42:1**	0,25 ± 0,09	0,28 ± 0,11	-0,11	0,048	-0,02	0,756	0,858	1,01	0,0028	0,0010	0,0011	0,0004	-0,0003
**PC aa C42:2**	0,22 ± 0,08	0,25 ± 0,12	-0,06	0,310	0,02	0,815	0,837	1,03	0,0020	0,0003	-0,0034	-0,0024	-0,0002
**PC aa C42:4**	0,21 ± 0,09	0,24 ± 0,14	-0,07	0,295	-0,02	0,774	0,825	1,22	0,0021	0,0005	-0,0040	-2,46E-05	0,0056
**PC aa C42:5**	0,44 ± 0,18	0,43 ± 0,18	0,05	0,370	0,05	0,441	0,846	1,06	-0,0007	-0,0016	-0,0052	0,0020	0,0040
**PC aa C42:6**	0,60 ± 0,19	0,60 ± 0,19	0,01	0,792	0,02	0,682	0,845	0,96	-0,0001	-0,0011	-0,0011	0,0065	0,0017
**PC ae C30:0**	0,38 ± 0,12	0,42 ± 0,18	-0,06	0,203	0,05	0,412	0,826	0,91	0,0023	0,0008	0,0030	0,0037	-0,0006
**PC ae C32:1**	2,52 ± 0,76	2,75 ± 1,03	-0,05	0,286	0,05	0,427	0,837	0,85	0,0020	0,0005	0,0029	0,0032	0,0026
**PC ae C32:2**	0,62 ± 0,20	0,68 ± 0,26	-0,07	0,173	0,04	0,540	0,809	0,85	0,0022	0,0005	0,0013	0,0011	-0,0013
**PC ae C34:0**	1,50 ± 0,52	1,59 ± 0,68	-0,02	0,659	0,05	0,412	0,827	0,77	0,0012	-2,66E-05	0,0017	0,0051	0,0004
**PC ae C34:1**	9,49 ± 2,88	10,08 ± 3,88	-0,03	0,609	0,06	0,305	0,759	0,74	0,0014	0,0001	0,0021	0,0041	0,0016
**PC ae C34:2**	10,93 ± 3,76	12,13 ± 5,73	-0,06	0,327	0,07	0,284	0,746	0,97	0,0020	0,0005	0,0011	-0,0042	-0,0045
**PC ae C34:3**	7,01 ± 2,51	8,03 ± 3,75	-0,09	0,130	0,09	0,169	0,618	1,02	0,0026	0,0010	0,0037	0,0010	0,0025
**PC ae C36:0**	0,85 ± 0,28	0,83 ± 0,31	0,05	0,347	0,12	0,048	0,330	0,85	-0,0006	-0,0017	-0,0056	-0,0056	-0,0048
**PC ae C36:1**	8,70 ± 2,71	9,69 ± 3,88	-0,07	0,209	0,02	0,743	0,857	0,97	0,0024	0,0006	-0,0002	0,0022	0,0030
**PC ae C36:2**	13,16 ± 4,55	14,72 ± 6,38	-0,08	0,163	0,05	0,398	0,817	0,97	0,0023	0,0007	0,0012	-0,0024	-0,0051
**PC ae C36:3**	7,20 ± 2,43	7,63 ± 3,43	-0,01	0,849	0,10	0,165	0,611	0,78	0,0012	-0,0002	-0,0004	-0,0047	-0,0048
**PC ae C36:4**	17,72 ± 6,55	17,25 ± 6,83	0,05	0,438	0,06	0,423	0,835	0,63	-0,0006	-0,0014	-0,0016	-0,0036	-0,0058
**PC ae C36:5**	11,86 ± 4,57	11,96 ± 4,70	1,30E-03	0,982	0,03	0,656	0,836	0,80	0,0002	-0,0007	0,0025	0,0061	0,0047
**PC ae C38:0**	2,25 ± 0,91	2,38 ± 1,08	-0,04	0,528	0,01	0,847	0,844	0,82	0,0011	-0,0003	-0,0020	0,0026	-0,0015
**PC ae C38:1**	0,78 ± 0,49	1,02 ± 0,90	-0,14	0,113	6,11E-04	0,995	0,692	1,43	0,0027	0,0011	-0,0046	-0,0027	0,0046
**PC ae C38:2**	2,02 ± 0,76	2,38 ± 1,37	-0,08	0,200	0,04	0,584	0,810	1,37	0,0026	0,0007	-0,0048	-0,0059	-0,0014
**PC ae C38:3**	4,14 ± 1,57	4,69 ± 2,49	-0,06	0,374	0,01	0,870	0,818	1,15	0,0021	0,0004	-0,0043	-0,0036	-0,0005
**PC ae C38:4**	12,77 ± 4,16	13,30 ± 4,79	-0,02	0,722	2,89E-04	0,996	0,496	0,70	0,0010	-0,0002	0,0024	0,0039	0,0013
**PC ae C38:5**	16,49 ± 5,72	16,61 ± 6,05	0,01	0,890	0,03	0,657	0,834	0,59	0,0002	-0,0008	0,0014	0,0020	-0,0003
**PC ae C38:6**	8,23 ± 3,39	8,30 ± 3,42	2,80E-03	0,964	0,02	0,750	0,859	0,51	0,0002	-0,0009	-0,0005	0,0010	-0,0019
**PC ae C40:1**	1,38 ± 0,45	1,53 ± 0,61	-0,07	0,206	3,98E-03	0,951	0,814	1,17	0,0023	0,0008	0,0040	0,0090	0,0066
**PC ae C40:2**	1,99 ± 0,66	2,14 ± 0,83	-0,05	0,374	0,02	0,752	0,858	0,90	0,0016	-0,0001	-0,0033	-0,0034	-0,0024
**PC ae C40:3**	1,27 ± 0,71	1,57 ± 1,12	-0,12	0,085	-0,03	0,706	0,856	1,42	0,0026	0,0009	-0,0052	-0,0047	0,0019
**PC ae C40:4**	2,31 ± 0,81	2,61 ± 1,16	-0,07	0,199	-0,03	0,700	0,854	1,05	0,0024	0,0006	-0,0023	-0,0017	0,0022
**PC ae C40:5**	3,40 ± 1,15	3,90 ± 1,77	-0,09	0,105	-0,04	0,524	0,800	1,13	0,0027	0,0009	-0,0009	0,0023	0,0050
**PC ae C40:6**	4,93 ± 2,04	5,37 ± 2,14	-0,09	0,141	-0,03	0,634	0,825	0,79	0,0017	0,0003	0,0021	0,0041	0,0007
**PC ae C42:1**	0,34 ± 0,12	0,37 ± 0,16	-0,06	0,310	0,01	0,861	0,822	1,00	0,0020	0,0005	4,07E-05	0,0052	0,0060
**PC ae C42:2**	0,58 ± 0,19	0,63 ± 0,26	-0,06	0,296	0,01	0,921	0,807	1,03	0,0020	0,0004	0,0007	0,0058	0,0068

**PC ae C42:3**	0,80 ± 0,27	0,90 ± 0,39	-0,08	0,150	0,02	0,806	0,838	0,95	0,0024	0,0006	-0,0005	-4,77E-06	0,0006
**PC ae C42:4**	0,85 ± 0,28	0,95 ± 0,39	-0,07	0,226	0,01	0,855	0,836	1,02	0,0023	0,0006	-0,0012	-0,0039	-0,0005
**PC ae C44:3**	0,14 ± 0,05	0,16 ± 0,08	-0,06	0,236	0,00	0,938	0,811	1,32	0,0020	0,0003	-0,0055	-0,0051	0,0035
**PC ae C44:4**	0,36 ± 0,11	0,39 ± 0,16	-0,05	0,390	0,05	0,383	0,806	0,82	0,0018	0,0003	-0,0001	-0,0031	0,0006
**PC ae C44:5**	1,55 ± 0,53	1,66 ± 0,68	-0,04	0,477	0,03	0,601	0,816	0,74	0,0014	0,0002	0,0022	-0,0013	-0,0005
**PC ae C44:6**	1,10 ± 0,39	1,20 ± 0,46	-0,07	0,226	0,02	0,768	0,824	0,84	0,0019	0,0005	0,0020	-0,0016	-0,0030
**Lyso-Phosphatidylcholines**													
**lysoPC a C16:0**	140,59 ± 44,80	145,53 ± 54,77	4,40E-03	0,935	0,05	0,403	0,821	0,62	0,0008	-0,0002	0,0022	0,0030	-0,0003
**lysoPC a C16:1**	3,66 ± 1,38	3,68 ± 1,57	0,04	0,555	0,08	0,261	0,728	1,07	0,0001	-0,0006	0,0037	0,0091	0,0012
**lysoPC a C17:0**	2,19 ± 0,79	2,72 ± 1,21	-0,18	0,004	-0,06	0,397	0,816	1,71	0,0041	0,0024	0,0090	0,0106	0,0048
**lysoPC a C18:0**	40,34 ± 13,54	43,02 ± 16,97	-0,03	0,564	0,01	0,828	0,839	0,65	0,0014	0,0002	0,0016	0,0010	-0,0016
**lysoPC a C18:1**	22,28 ± 7,28	25,89 ± 10,34	-0,11	0,057	-1,67E-03	0,980	0,774	1,59	0,0032	0,0018	0,0090	0,0113	0,0070
**lysoPC a C18:2**	25,85 ± 9,53	31,23 ± 15,39	-0,12	0,061	0,04	0,581	0,811	1,44	0,0033	0,0020	0,0077	0,0035	-0,0001
**lysoPC a C20:3**	2,32 ± 0,77	2,34 ± 1,08	0,06	0,345	0,09	0,213	0,672	0,56	0,0002	-0,0007	0,0003	0,0009	-0,0051
**lysoPC a C20:4**	6,40 ± 2,45	6,85 ± 3,15	-0,03	0,606	-0,02	0,832	0,840	1,25	0,0013	0,0006	0,0071	0,0104	0,0022
**lysoPC a C28:1**	0,39 ± 0,12	0,43 ± 0,17	-0,09	0,074	0,01	0,862	0,818	1,06	0,0026	0,0009	-0,0013	-0,0027	-0,0026
**Sphingomyelins**													
**SM (OH) C14:1**	7,24 ± 2,52	8,09 ± 3,12	-0,10	0,072	0,01	0,898	0,821	0,96	0,0024	0,0009	0,0033	0,0010	-0,0014
**SM (OH) C16:1**	3,93 ± 1,38	4,36 ± 1,64	-0,09	0,073	-5,66E-04	0,992	0,749	0,92	0,0023	0,0009	0,0034	0,0017	0,0014
**SM (OH) C22:1**	15,56 ± 4,73	16,08 ± 5,49	-0,01	0,795	0,06	0,269	0,734	0,69	0,0008	-0,0005	-0,0004	-0,0043	-0,0031
**SM (OH) C22:2**	12,20 ± 4,03	13,53 ± 4,88	-0,09	0,078	0,01	0,828	0,839	1,00	0,0024	0,0009	0,0045	0,0032	0,0024
**SM (OH) C24:1**	1,52 ± 0,49	1,58 ± 0,56	-0,02	0,639	0,07	0,254	0,722	0,79	0,0009	-0,0003	-0,0003	-0,0053	-0,0046
**SM C16:0**	115,05 ± 32,93	122,45 ± 38,23	-0,05	0,277	0,05	0,323	0,774	0,81	0,0017	0,0002	0,0023	-4,97E-05	0,0027
**SM C16:1**	18,01 ± 5,33	18,68 ± 5,70	-0,03	0,540	0,04	0,429	0,838	0,61	0,0010	-0,0003	0,0009	-0,0013	-0,0006
**SM C18:0**	27,77 ± 8,62	27,86 ± 9,35	0,01	0,771	0,06	0,276	0,738	0,58	0,0001	-0,0010	-0,0018	-0,0038	0,0008
**SM C18:1**	13,15 ± 4,20	13,42 ± 4,54	-0,01	0,892	0,03	0,525	0,800	0,49	0,0005	-0,0006	-0,0002	-0,0018	0,0005
**SM C20:2**	0,38 ± 0,15	0,42 ± 0,18	-0,11	0,067	0,04	0,567	0,805	0,99	0,0023	0,0010	0,0031	-0,0018	-0,0008
**SM C24:0**	24,41 ± 7,34	23,47 ± 7,55	0,06	0,227	0,13	0,027	0,225	0,98	-0,0010	-0,0020	-0,0050	-0,0092	-0,0043
**SM C24:1**	60,24 ± 19,93	61,79 ± 19,87	-0,02	0,666	0,07	0,234	0,697	0,62	0,0006	-0,0006	-0,0003	-0,0024	0,0029
**SM C26:0**	0,23 ± 0,07	0,23 ± 0,09	4,20E-03	0,939	0,11	0,081	0,429	0,77	0,0006	-0,0005	-0,0003	-0,0057	-0,0041
**SM C26:1**	0,53 ± 0,23	0,54 ± 0,21	-0,02	0,655	0,06	0,346	0,782	0,49	0,0003	-0,0007	-0,0016	-0,0033	-0,0005

[1] P values and regression coefficients (ß) derived from a linear regression analysis of the log-transformed metabolite concentrations in 230 study participants (115 FLD cases, 115 controls). All models were also adjusted for sex, age, body mass index, triglyceride level, hemoglobin, glycated hemoglobin and GGT.

[2] p_{adj} denotes the p value adjusted for multiple testing by the Westfall and Young Step-Down MaxT procedure with 10,000 permutations.

[3] Partial least-squares discriminant analysis (PLS-DA) was based upon 230 study participants, whereas missing values were imputed with corresponding sample means for cases or controls. VIP (Variable Importance in the Projection) scores were calculated on the basis of the first five components from the PLS-DA. Loadings represent the coefficients that define a linear combination of the first five components of the PLS-DA respectively.

[4] Linear regression analysis was based upon 229 (leucine), 219 (histamine), 221 (SDMA) and 214 (taurine) individuals only, respectively, because of missing data.
