# Supplementary material for: Diagnosing Fatty Liver Disease: A Comparative Evaluation of Metabolic Markers, Phenotypes, Genotypes and Established Biomarkers
Source: PLoS One. 2013 Oct 9;8(10):e76813. doi: 10.1371/journal.pone.0076813 (PMC3793954; doi:10.1371/journal.pone.0076813)
Supplement: Material S1 — SNP genotyping, imputation and quality control. (PDF) [file pone.0076813.s004.pdf]

Supplementary Material to:

Diagnosing fatty liver disease: a comparative evaluation of metabolic markers, phenotypes, genotypes and established biomarkers

Sabine Siegert, Zhonghao Yu, Rui Wang-Sattler, Thomas Illig, Jerzy Adamski, Jochen Hampe, Susanna Nikolaus, Stefan Schreiber, Michael Krawczak, Michael Nothnagel, Ute Nöthlings

M1: SNP genotyping, imputation and quality control

Information on single nucleotide polymorphisms (SNPs) related to fatty liver disease (FLD) was obtained from available genotype data or rather by imputation. Briefly, for 225 (115 cases, 110 controls) out of the included 230 samples, genome-wide genotype data of 934,968 single nucleotide polymorphisms (SNPs) from the Affymetrix® Genome-Wide Human SNP Array 6.0 were available from previous studies (1-2). Thirty-eight individuals were excluded from further analysis either due to being a genetic "outlier" of presumed non-European ancestry (n=4), low genotyping call rate (<98%; n=33) or excess homozygosity/heterozygosity (n=1), leaving 187 (n=91 cases, 96 controls) samples for further analyses. All sex assignments could be confirmed by reference to the proportion of heterozygous SNPs on the X-chromosome. SNPs showing a low call-rate (<98% in either cases or controls), a low minor allele frequency (MAF<2% in either cases or controls) or an excessive deviation from Hardy Weinberg equilibrium (HWE) in the controls ($p<10^{-4}$) were excluded (n=275,989; 30%), leaving 658,979 SNPs for further analysis. The described quality checks were performed with R (v. 2.14.1) (3) or PLINK (v. 1.07) (4), as appropriate. Genotype information was available for some of selected susceptibility SNPs (rs1801121, rs780094, rs1800795, rs767870), while genotypes of the remaining candidate SNPs were imputed with Beagle (v. 3.3) (5) based exclusively on the quality-controlled genotypes and with an interval of 1 MB (mega base pairs) around the candidate SNPs, respectively. We used the panel of 283 Europeans sequenced by the 1000 Genomes Project (6) (publicly available at http://faculty.washington.edu/browning/beagle/, downloaded 2010/12/14) as imputation basis, covering approximately 12 million SNPs. Four SNPs (rs12137855, rs2854116, rs2228603, rs738409) that had an imputation score $r^2<0.8$ and/ or a posterior probability <90% for the most likely genotype in at least 90% of all 187 samples combined were excluded, leaving ten SNPs in the genetic analysis.

References

1. Lascorz J, Forsti A, Chen B, et al. Genome-wide association study for colorectal cancer identifies risk polymorphisms in German familial cases and implicates MAPK signalling pathways in disease susceptibility. Carcinogenesis 2010; 31(9): 1612-9.
2. Nothnagel M, Ellinghaus D, Schreiber S, Krawczak M, Franke A. A comprehensive evaluation of SNP genotype imputation. Hum Genet 2009; 125(2): 163-71.
3. R Development Core Team. R: A language and environment for statistical computing. 2010.
4. Purcell S, Neale B, Todd-Brown K, et al. PLINK: a tool set for whole-genome association and population-based linkage analyses. Am J Hum Genet 2007; 81(3): 559-75.
5. Browning SR, Browning BL. Rapid and accurate haplotype phasing and missing-data inference for whole-genome association studies by use of localized haplotype clustering. Am J Hum Genet 2007; 81(5): 1084-97.
6. Consortium TGP. A map of human genome variation from population-scale sequencing. Nature 2010; 467(7319): 1061-73.
