# Supplementary figures and images for: Diagnosing Fatty Liver Disease: A Comparative Evaluation of Metabolic Markers, Phenotypes, Genotypes and Established Biomarkers
Source: PLoS One. 2013 Oct 9;8(10):e76813. doi: 10.1371/journal.pone.0076813 (PMC3793954; doi:10.1371/journal.pone.0076813)

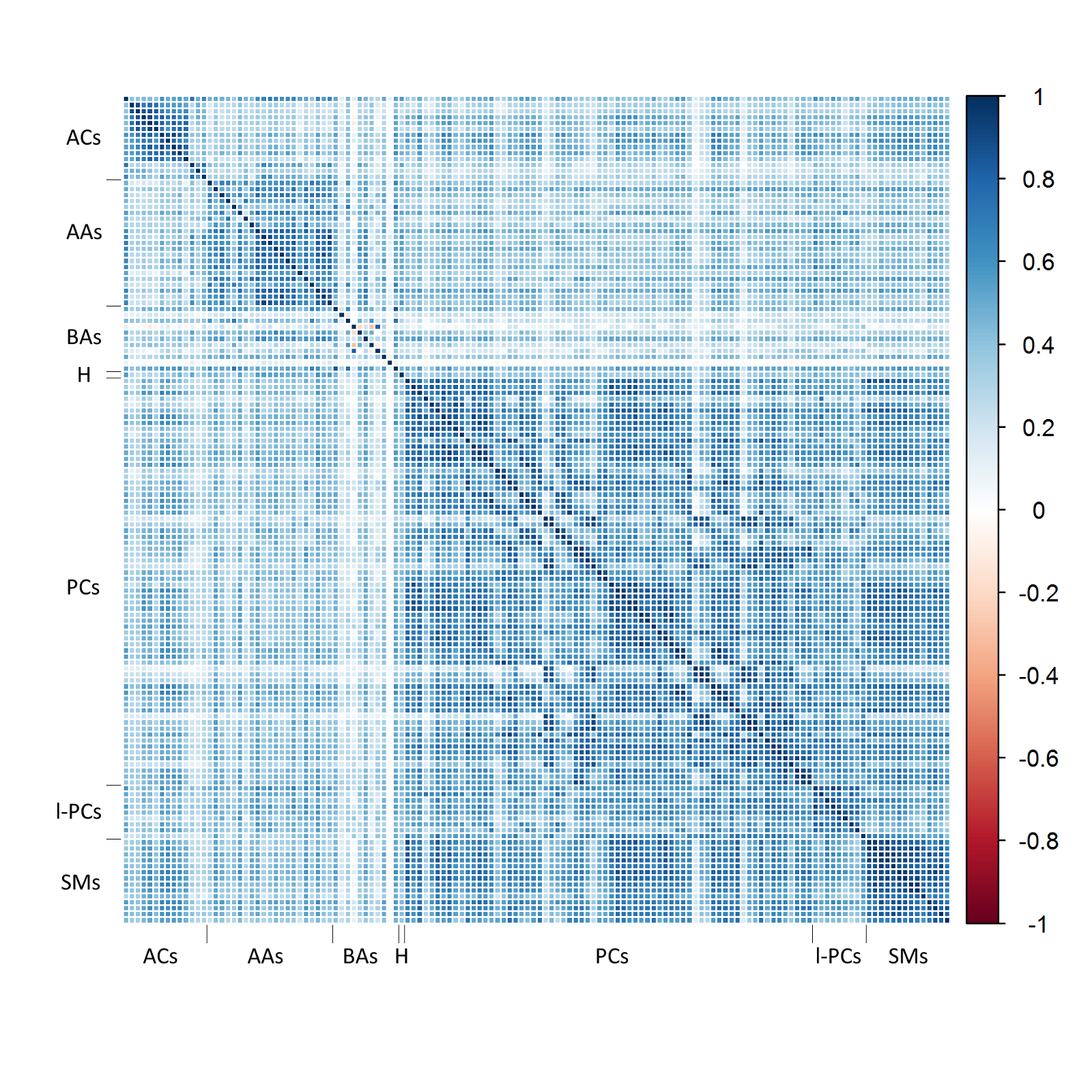

Supplement: Figure S1 — Correlation structure among the 138 metabolites under study. The graphical display was based on Pearson’s correlation coefficients. ACs: acylcarnitines (n=14), AAs: amino acids (n=21), BAs: biogenic amines (n=11), H: hexose (n=1), PCs: phosphatidylcholines (n=69), l-PCs: lyso-phosphatidylcholines (n=9), SMs: sphingomyelins (n=14) (TIFF) [file pone.0076813.s005.tiff]
